# Rescuing tri-heteromeric NMDA receptor function: the potential of pregnenolone-sulfate in loss-of-function GRIN2B variants

**DOI:** 10.1007/s00018-024-05243-x

**Published:** 2024-05-25

**Authors:** Shai Kellner, Shai Berlin

**Affiliations:** Dept. of Neuroscience, Ruth and Bruce Rappaport Faculty of Medicine, Technion-Israel Institute of Technology, 1 Efron Bat Galim, Haifa, 3525433 Israel

**Keywords:** NMDAR, GRIN, GRINopathy, Variant, Pregnenolone-sulfate, GRIN2B, Loss-of-function, Tri-heteromers

## Abstract

N-methyl-D-aspartate receptors (NMDARs emerging from *GRIN* genes) are tetrameric receptors that form diverse channel compositions in neurons, typically consisting of two GluN1 subunits combined with two GluN2(A-D) subunits. During prenatal stages, the predominant channels are di-heteromers with two GluN1 and two GluN2B subunits due to the high abundance of GluN2B subunits. Postnatally, the expression of GluN2A subunits increases, giving rise to additional subtypes, including GluN2A-containing di-heteromers and tri-heteromers with GluN1, GluN2A, and GluN2B subunits. The latter  emerge as the major receptor subtype at mature synapses in the hippocampus. Despite extensive research on purely di-heteromeric receptors containing two identical *GRIN* variants, the impact of a single variant on the function of other channel forms, notably tri-heteromers, is lagging. In this study, we systematically investigated the effects of two de novo* GRIN2B* variants (G689C and G689S) in pure, mixed di- and tri-heteromers. Our findings reveal that incorporating a single variant in mixed di-heteromers or tri-heteromers exerts a dominant negative effect on glutamate potency, although ‘mixed’ channels show improved potency compared to pure variant-containing di-heteromers. We show that a single variant within a receptor complex does not impair the response of all receptor subtypes to the positive allosteric modulator pregnenolone-sulfate (PS), whereas spermine completely fails to potentiate tri-heteromers containing GluN2A and -2B-subunits. We examined PS on primary cultured hippocampal neurons transfected with the variants, and observed a positive impact over current amplitudes and synaptic activity. Together, our study supports previous observations showing that mixed di-heteromers exhibit improved glutamate potency and extend these findings towards the exploration of the effect of Loss-of-Function variants over tri-heteromers. Notably, we provide an initial and crucial demonstration of the beneficial effects of *GRIN2B*-relevant potentiators on tri-heteromers. Our results underscore the significance of studying how different variants affect distinct receptor subtypes, as these effects cannot be inferred solely from observations made on pure di-heteromers. Overall, this study contributes to ongoing efforts to understand the pathophysiology of *GRINopathies* and provides insights into potential treatment strategies.

**Supplementary Information:**

The online version contains supplementary material available at 10.1007/s00018-024-05243-x.

## Introduction

N-methyl-D-aspartate receptors (NMDARs or GluNRs) are ionotropic non-selective cation channels, composed of multiple subunits derived from seven different genes (*GRIN1, GRIN2A-D, GRIN3A-B*) [1, 2]. Typically, functional NMDARs consist of two obligatory GluN1 subunits (glycine-binding) in conjunction with two glutamate-binding GluN2 subunits [3]. This arrangement gives rise to a diverse array of receptor subtypes expressed on neuronal membranes, which undergo dynamic changes during development and in response to synaptic activity [1, 2, 4]. Previously, it was assumed that NMDARs primarily consisted of two GluN1 subunits and two identical GluN2 subunits (di-heteromers), such as the prevalent di-heteromeric GluN1/GluN2B-receptor that is highly abundant in the forebrain at prenatal stages [2, 5–7]. However, emerging evidence suggests that functional NMDARs can also be composed of two GluN1 subunits and two distinct GluN2 subunits, denoted tri-heteromers [8]. Indeed, the postnatal increase in the expression of the GluN2A subunit in various brain regions enables the formation of di-heteromers solely containing GluN2A (i.e., GluN1 + GluN1 + GluN2A + GluN2A), as well as tri-heteromers containing a single GluN2A-subunit mixed with other subunits, in particular GluN2B [6]. While the prevalence of tri-heteromers has been a topic of debate for some time (discussed in [7]), recent findings suggest that GluN1/GluN2A/GluN2B tri-heteromers are the predominant receptor subtype in several brain regions, particularly the hippocampus [7–9].

In the central nervous system, NMDARs play essential roles in many process, such as synaptic maturation, dendritogenesis, synaptic plasticity, and in processes of learning and memory, to name a few [1, 2, 7, 10–13]. Thus, disruption of NMDAR function and/or expression is expected to adversely affect brain development and function, such as in the case of *GRIN* variants [14]. It is now well appreciated that *GRIN* variants, although relatively rare (~ 1:5000 [15, 16]), underlie a wide range of encephalopathies, among them neurodevelopmental disorders, severe mental retardation, and epilepsies [14, 17–23]. Systematic screenings of pediatric patients have revealed numerous (thousands [17, 24]) of inherited or de novo* GRIN* variants, with significantly higher incidence in the genes encoding for the more abundant subunits in the brain, namely GluN2A and GluN2B-subunits (46% and 38%, respectively) [17, 21, 23–28]. Variants (whether missense, nonsense, or frameshift mutations) occur sporadically throughout the entire length of the genes, with each variant imposing very distinct effects over receptor function and/or expression.

To date, the majority of characterizations, including our own efforts [17], have mainly concentrated on evaluating variants within the context of pure di-heteromers (e.g., [14, 18, 21, 25, 29–31]), followed by explorations of variants over mixed di-heteromers, namely receptors containing two GluN1 subunits paired with a *wt* and a variant of the same GluN2 subunit, though to a much lesser extent (see Table 1) [19, 21, 23, 32, 33]. These datasets have revealed that amino terminal and ligand binding domains (NTD and LBD, respectively) are more likely to be associated with a Loss-of-Function (LoF) functional phenotypes [27, 34], whereas transmembrane-domain or inter-domain-linker variants are more likely to be associated with Gain-oF (GoF) attributes instead [23, 27, 34]. Nevertheless, exceptions to these rules are emerging [27]. We have previously found that pure di-heteromers containing two GluN2B-variants indeed instigated the ‘expected’ LoF phenotype, however the magnitude of this effect could not have been anticipated. We showed that the two variants explored engendered the most severe reductions in glutamate potency to be reported for GluN2B-containing receptors (~ 2000-fold reduction in glutamate potency) [17]. We also showed we could not have anticipated the variants’ overlapping, but also unique, effects over other features of the pure di-heteromeric receptor such as proton sensitivity, expression levels, and responsiveness to selective potentiators [17]. Thus, it stands that there are no reliable predictive methods to determine a priori the impact of variants over different aspects of pure and mixed di-heteromers. Importantly, whether a single variant would similarly impact the function of other receptor subtypes—tri-heteromers in particular—remains the least explored. In fact, to the best of our knowledge, only three reports have explored the impact of *GRIN* variants in the context of tri-heteromeric NMDARs (LoF/GoF *GRIN2A* and *GRIN2B* variants [8, 10, 20]).

**Table 1:** Summary of the glutamate potency for mixed di- and tri-heteromeric NMDAR variants

Variant	Type	*wt* Di-Heteromeric EC_50_ (μM)	Mixed-Di-Heteromeric EC_50_ (μM)	Pure variant Di-Heteromeric EC_50_ (μM)	Potency ratio	References
GluN2A-H485A	LOF	4.2	220	1060	4.8	[8]
GluN2A-P552R	GOF	3.9	1.2	0.64	3.3	[32]
GluN2A-S644G	GOF	4.5	0.7	0.05	6.4	[19]
GluN2A-T690A	LOF	4.2	660	2700	4.1	[8]
GluN2A-D731N	LOF	5.7	6451	30,000	4.7	[33]
GluN2A-L812M	GOF	4.0	1.0	0.39	4.0	[39]
GluN2A-M817V	GOF	3.6	1.1	0.47	3.3	[91]
GluN2B-E413G	LOF	0.9	21	82	3.9	[21]
GluN2B-C461F	LOF	1.5	61	290	4.8	[21]
GluN2B-H486A	LOF	1.6	75	460	6.1	[8]
GluN2B-G689C	LOF	0.8	368.1	1700	4.6	Current paper
GluN2B-G689S	LOF	0.8	760.8	2800	3.7	Current paper

Variant	Type	*wt* Tri-Heteromeric EC_50_ (μM)	Mixed tri-heteromeric EC_50_ (μM)	Pure variant di-heteromeric EC_50_ (μM)	Potency ratio	
GluN2A-*wt/* GluN2B-G543R	GOF	2.2	1.9	0.22	1.2	[10]
GluN2A-*wt/* GluN2B-A639V	GOF	2.2	0.58	0.28	3.8	[10]
GluN2A-*wt/* GluN2B-G689C	LOF	1.5	503	1700	3.4	Current paper
GluN2A-*wt/* GluN2B-G689S	LOF	1.5	937	2800	3.0	Current paper
GluN2A-*wt/* GluN2B-M818T	GOF	2.2	0.96	0.37 [25]	2.3	[10]
GluN2A-*wt/* GluN2B-A819T	GOF	2.2	0.71	0.58 [25]	3.1	[10]
GluN2A-E551K/ GluN2B-*wt*	GOF	2.2	0.70	0.47	3.1	[10]
GluN2A-P552R/ GluN2B-*wt*	GOF	2.2	0.56	0.37 [32]	3.9	[10]
GluN2A-S644G/ GluN2B-*wt*	GOF	2.2	0.49	0.18 [19]	4.5	[10]
GluN2A-L649V/ GluN2B-*wt*	GOF	2.2	0.36	0.05	6.1	[10]
GluN2A-L812M/ GluN2B-*wt*	GOF	2.2	0.84	0.39 [39]	2.6	[10]
GluN2A-M817V/ GluN2B-*wt*	GOF	2.2	0.75	0.47 [91]	2.9	[10]

Variant	Type	*wt* Tri-Heteromeric EC_50_ (μM)	Mixed Tri-Heteromeric EC_50_ (μM)	Double Variant Tri-Heteromeric EC_50_ (μM)	Potency ratio	
GluN2A-*wt*/ GluN2B-H486A	LOF	2.6	140	730	5.2	[8]
GluN2A-H485A/ GluN2B-*wt*	LOF	2.6	210	730	3.5	[8]

Potency ratio was calculated as: Highest EC_50_/[mixed di or tri-heteromeric EC_50_]

To address this gap, we conducted a detailed examination of the impact of the two severe LoF *GRIN2B* variants (GluN2B-G689C and GluN2B-G689S [17]) in the context of pure and mixed di- and tri-heteromeric channels. To regulate channel stoichiometry, we employed a unique ER-retention technique [8], to ensure the expression of a desired receptor population at the cell membrane (see Methods). Our findings reveal that the incorporation of a single GluN2B-variant within mixed di- or tri-heteromeric channels similarly leads to a significant reduction in glutamate potency. However, these reductions are not as drastic as those observed in purely di-heterometric receptors containing two copies of the variants. Moreover, mixed di-heteromers exhibit normal pH-sensitivity and, consequently, spermine-responsivity (a GluN2B-selective potentiator), whereas spermine is entirely inefficient in potentiating pure di-heteromers containing the GluN2B-variants. Interestingly, the variants do not disrupt the ability of various channel types to respond to pregnenolone-sulfate (PS), a GluN2A and GluN2B-selective positive allosteric modulator (PAM). Application of PS partially rescues the amplitude of NMDAR-dependent currents in neurons expressing the variants individually.

Our results emphasize the unique properties of pure and mixed heteromers containing two unique GluN2B variants studied here (G689C or G689S), in combination with GluN2A-*wt* and GluN2B-*wt* subunits. These underscore the challenge of predicting a priori whether and how each variant would affect features of mixed di- and tri-heteromeric forms of the receptors. Importantly, we find that PS has a positive outcome over variant receptors, suggesting its potential as a treatment for LoF variants, even in the case of extremely deleterious variants such as GluN2B-G689C and GluN2B-G689S.

## Results

### Loss-of-Function variants in GluN2B instigate a dominant-negative effect over mixed di-heteromers

**Fig. 1:**
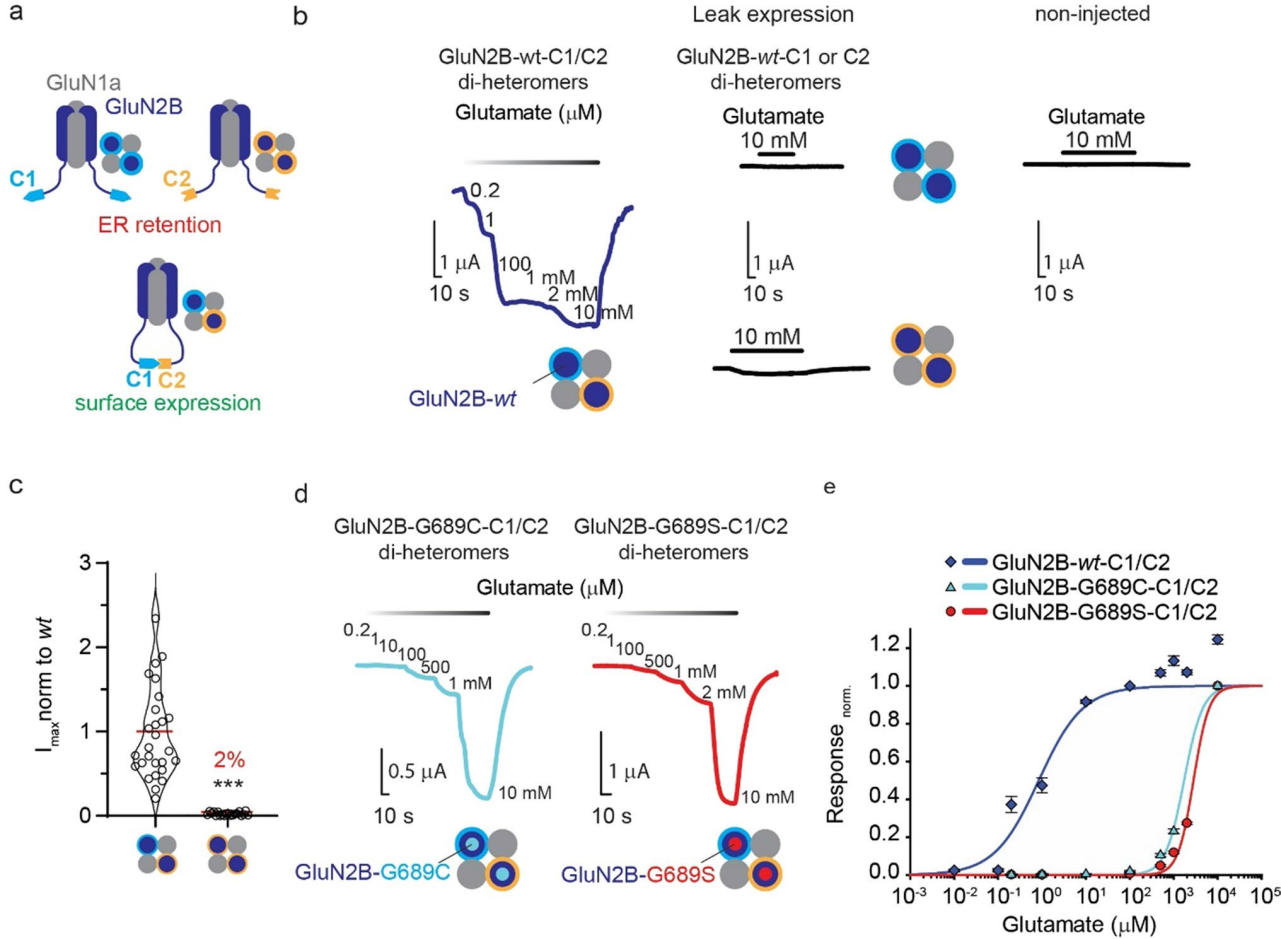
Selective expression of NMDAR-subtypes at the surface of *Xenopus Laevis* oocytes. **a** Cartoon depiction of a pure di-heteromeric receptor composed of two GluN1-1a-*wt* subunits (gray) assembled with two GluN2B-subunits (dark blue). Left: receptors containing two GluN2B-subunits tagged with C1 tails (cyan carboxy-termini outlines dark blue circle) or with C2 tails (orange carboxy-termini outlines dark blue circle) are retained in ER, whereas receptors containing two GluN2B subunits with C1- (cyan) and C2-termini (orange) are trafficked to the membrane (bottom cartoon). **b** Representative traces showing glutamate-dependent currents recorded from oocytes co-injected with GluN1-1a-*wt* with GluN2B-*wt*-C1 and GluN2B-*wt*-C2, denoted GluN2B-*wt*-C1/C2 (left, dark blue trace); oocytes injected with GluN1-1a-*wt* and GluN2B-*wt*-C2 mRNA only (middle; leak); non-injected oocytes (right). Glutamate (and glycine) application is noted by black or gradient bars above traces. **c** Summary of maximal currents (I_max_) comparing GluN2B-*wt*-C1/C2 (N = 4, n = 34) and leak currents (from oocytes expressing GluN2B-wt-C2; N = 4, n = 20), 72 hrs after mRNA injection; average highlighted in red. **d** Representative traces showing glutamate dose–response currents recorded from oocytes co-expressing GluN1a-*wt* with GluN2B-G689C-C1/C2 (cyan trace; GluN2B-G689C variant is denoted by a small filled cyan circle within the GluN2B subunit- dark blue) or with GluN2B-G689S-C1/C2 (red trace; GluN2B-G689S- red filled circles) Glutamate (and glycine) application is noted by gradient above traces. **e** Summary of dose–response curves for GluN1-1a-*wt* + GluN2B-*wt*-C1/C2 (blue), GluN1-1-a-*wt* + GluN2B-G689C-C1/C2 (cyan) and GluN1-1a-*wt* + GluN2B-G689S-C1/C2 (red). Data points represent mean ± SEM. Dose response curves were generated by fitting the normalized data using the adapted Hill equation (see Methods). For **(c)** Significance was assessed by Mann–Whitney test. ***p < 0.001

We have previously identified two analogous de novo variants in the LBD of GluN2B at residue G689 (GluN2B-G689C and GluN2B-G689S [17]). Both human variants engender a dramatic reduction in glutamate potency (*apparent* glutamate affinity or EC_50_) in purely di-heteromeric receptors containing two variant GluN2B-subunits co-assembled with two GluN1-1a-*wt* subunits (for brevity, we omit mention of GluN1-1a subunits hereafter). However, owing to the heterozygous-nature of most *GRINopathies*, variant GluN2B-subunits may also multimerize with GluN2B-*wt* subunits to form mixed di-heteromers, which likely better reflects the prenatal expression pattern in patients [7, 35]. Notably, this scrutiny is relatively less common in the field of *GRIN* variants (e.g., [10, 19, 23, 33, 35]) (see Table 1 and discussion). To try to examine the effect of the variants in the context of mixed di-heteromeric receptors, we sought means to control channel stoichiometry at the membrane which led us to explore an established method for selective cell-surface-expression of NMDARs [8, 36]. Briefly, we obtained GluN2A and 2B-subunits that were tagged at their carboxy termini with leucine zipper motifs from GABA_B1_ or GABA_B2_ along ER-retention motifs (denoted C1 and C2, respectively) (Methods), which then permits predominant (~ 95%) surface expression of receptors composed of C1- and C2-tagged GluN2-subunits (denoted C1/C2), whereas C1/C1 or C2/C2-containing channels are mainly retained in the ER (Fig. 1a and Supplementary 1a–c) [8].

We inserted point mutations within the rat GluN2B-*wt*-C1 or C2-tagged clones to produce GluN2B-G689C and GluN2B-G689S subunits tagged with C1 or C2, co-expressed these along the rat GluN1-1a-*wt* subunit in *Xenopus* oocytes, and assessed activity of different receptor compositions via two-electrode voltage clamp (TEVC) (Methods) [37, 38]. We initially co-expressed GluN2B-*wt*-C1 with GluN2B-*wt*-C2 and find that these *wt*, albeit tagged, receptors express at the membrane and yield specific glutamate-dependent currents (Fig. 1b, c), whereas the sole expression of just one subunit-type, namely GluN2B-*wt*-C1 or GluN2B-*wt*-C2 with GluN1-1a, yields negligeable (~ 2%) glutamate-currents (denoted leak) when compared to oocytes expressing both clones. Importantly, leak currents were mainly noticeable when oocytes were exposed to saturating glutamate concentrations (10 mM, Fig. 1b, c, Supplemantary 1c, cyan-cyan). We compared the potency of purely di-heteromeric channels composed of GluN2B-G689C-C1/C2 and GluN2B-G689S-C1/C2-channels to that of GluN2B-*wt-*C1/C2 receptors and find that variant receptor (GluN2B-G689C and GluN2B-G689S) exhibit ~ 2000- and > 3000-fold increases in the EC_50_ values, respectively, on par with our previous observations with the non-tagged human variant receptors (Fig. 1d, e and Table 2)[17].

**Table 2:** Summary of pharmacological profiling for G689C and G689S mixed di- and tri-heteromeric receptors in *Xenopus Leavis* oocytes

Group	Glutamate EC_50_ (adapted from[16])	Glutamate EC_50_ (here)	Hill coefficient (n_H_)	Spermine Potentiation (Fold change)	PS Potentiation (Fold change)	proton IC_50_
Purely di-heteromeric GluN2B-*wt*	1.4 ± 0.1 μM (N = 3, n = 43)	0.8 $\pm$ 0.4 μM (N = 6, n = 38)	0.86 $\pm$ 0.29 (N = 6, n = 38)	1.97 $\pm$ 0.56 (N = 3, n = 25)	3.03 $\pm$ 0.13 (N = 3, n = 16)	7.26 ± 0.02 (N = 1, n = 11) Non-tagged 7.21 ± 0.03 (N = 1, n = 7)
Purely di-heteromeric GluN2B-G689C	1.5 ± 0.2 mM *** (N = 2, n = 31)	1.7 $\pm$ 0.2 mM *** (N = 2, n = 13)	2.04 $\pm$ 0.28 * (N = 2, n = 13)	–	3.96 $\pm$ 0.14 *** (N = 3, n = 18)	6.97 ± 0.05 *** (N = 1, n = 8)
Purely di-heteromeric GluN2B-G689S	2.5 ± 0.4 mM *** (N = 2, n = 23)	2.8 $\pm$ 0.2 mM *** (N = 2, n = 9)	2.45 $\pm$ 0.41 ** (N = 2, n = 9)	–	4.68 $\pm$ 0.19 *** (N = 3, n = 23)	6.85 ± 0.05 *** (N = 1, n = 8)
Mixed di-heteromeric GluN2B-*wt*/GluN2B-G689C		368.1 $\pm$ 55.6 μM *** (N = 6, n = 54)	0.75 $\pm$ 0.08 n.s (N = 6, n = 54)	1.78 $\pm$ 0.51 * (N = 2, n = 20)	2.78 $\pm$ 0.15 n.s. (N = 3, n = 13)	7.21 ± 0.03 n.s (N = 1, n = 14)
Mixed di-heteromeric GluN2B-*wt*/GluN2B-G689S		760.8 $\pm$ 64.6μM *** (N = 1, n = 13)	1.02 $\pm$ 0.10 n.s (N = 1, n = 13)	1.61 $\pm$ 0.03 *** (N = 2, n = 24)	2.99 $\pm$ 0.10 n.s (N = 3, n = 20)	7.18 ± 0.03 n.s. (N = 1, n = 13)
Tri-heteromeric GluN2A-*wt/*GluN2B-*wt*		1.5 $\pm$ 0.7 μM (N = 3, n = 33)	0.77 $\pm$ 0.22 (N = 3, n = 33)	0.73 $\pm$ 0.02 (N = 2, n = 12)	1.87 $\pm$ 0.04 (N = 4, n = 30)	–
Tri-heteromeric GluN2A-*wt*/GluN2B-G689C		502.9 $\pm$ 84.6 μM *** (N = 1, n = 10)	0.85 $\pm$ 0.14 n.s (N = 1, n = 10)	0.70 $\pm$ 0.03 n.s (N = 1, n = 10)	2.12 $\pm$ 0.10 n.s (N = 3, n = 13)	–
Tri -heteromeric GluN2A-*wt*/GluN2B-G689S		936.9 $\pm$ 91.2 μM *** (N = 1, n = 11)	1.06 $\pm$ 0.13 n.s (N = 1, n = 11)	0.75 $\pm$ 0.02 n.s (N = 2, n = 19)	2.02 $\pm$ 0.06 n.s (N = 2, n = 13)	–

Spermine was used at 200 μM, PS- 100 μM. EC_50_, IC_50_ and n_H_ values were extracted by fitting the data to adapted Hill equations Eq. (1) and Eq. (2) (see methods). (*N*) indicates individual experiments, whereas (*n*) indicates number of cells. Significance was assessed by one-way ANOVA with Tukey post hoc test compared to control group (by shades). *p < 0.05; **p < 0.01; ***p < 0.001; *n.s.* not significant

**Fig. 2:**
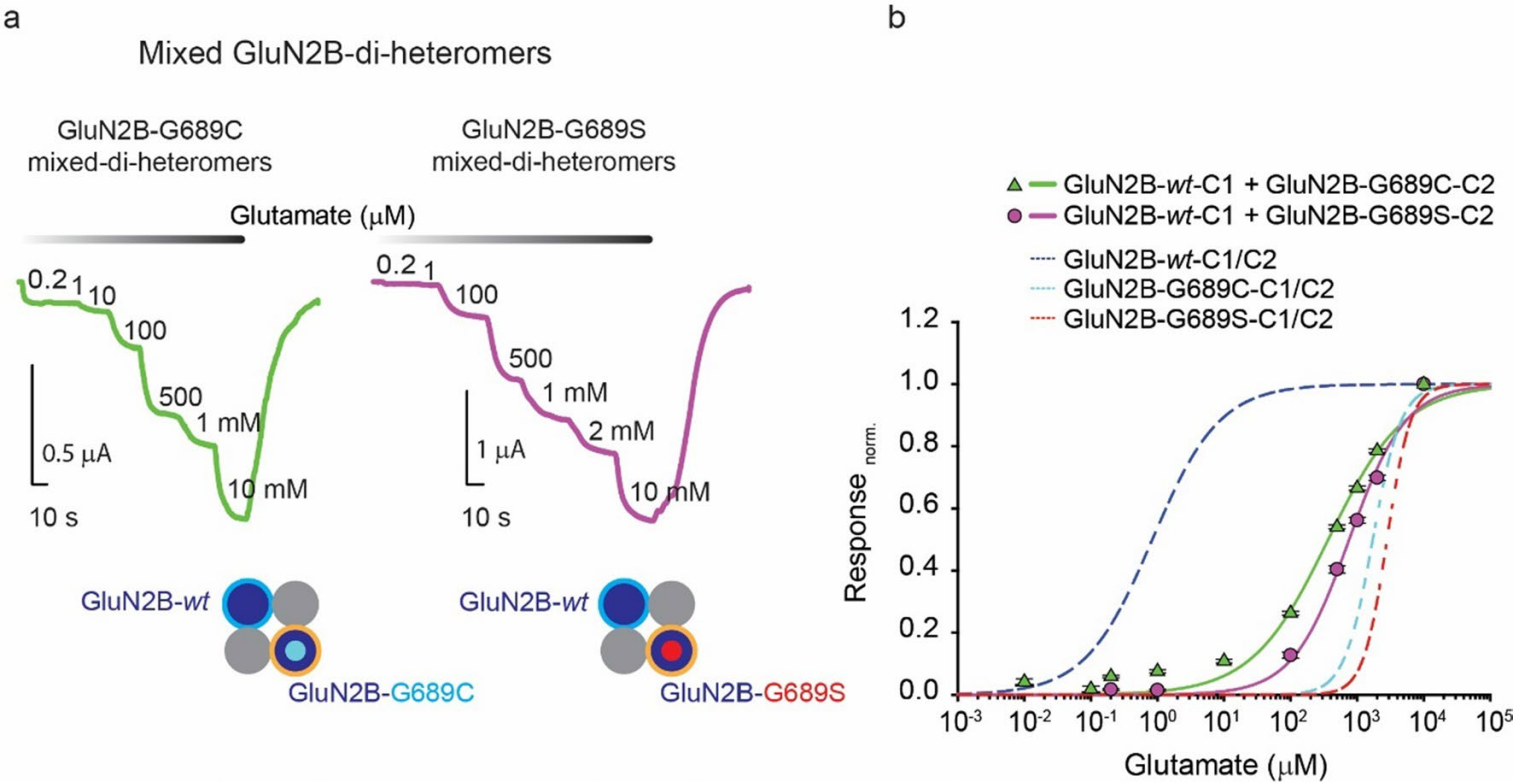
GluN2B-variants reduce glutamate potency of *mixed* di-heteromeric receptors. **a** Representative traces from oocytes expressing *mixed* GluN2B di–heteromers composed of GluN1-1a-*wt* with GluN2B-*wt*-C1 and GluN2B-G689C-C2 (lime trace) or with GluN2B-G689S-C2 (purple trace), in response to increasing glutamate concentrations. Glutamate (and glycine) application is noted by white-to-black gradient bar above traces. Glutamate concentrations (in μM) are noted next to current steps. Concentrations in mM are explicitly noted next to last steps. **b** Summary of dose–response curves for *mixed* GluN2B-di–heteromers, colored-coded as in (**a**). Dashed lines depict dose–response curves for purely di-heteromeric receptors shown in Fig. 1e- GluN2B-*wt*-C1/C2 (blue), GluN2B-G689C-C1/C2 (cyan) and GluN2B-G689S-C1/C2 (red). Data points represent mean ± SEM. Dose response curves were generated by fitting the normalized data using the adapted Hill equation (see Methods)

To explore the effect of single variants within a mixed di-heteromeric channel, we co-expressed GluN2B-*wt*-C1 with GluN2B*-C2 (*: G689C or G689S variants) along GluN1-1a-*wt*. Expectedly, mixed GluN2B-di-heteromeric channels showed major increases in the EC_50_ values, with GluN2B-G689S-containing channels displaying a more severe rightward shift (Fig. 2a, b, Table 2); highly consistent with this variant’s effect over pure di-heteromers (see Fig. 1 and [17]). Nevertheless, both mixed di-heteromeric channels showed ~ four-fold higher (i.e., improved) glutamate potencies compared to purely di-heteromeric variant channels (Fig. 2b, solid vs. dashed plots; see summary in Table 2; Table 1 for glutamate potency ratio). Given the very minimal leak expression (~ 2%) (see Fig. 1c), significant changes in glutamate potencies (EC_50_) cannot be accounted for by other channels compositions. Thus, we can ascertain that a single variant within a mixed di-heteromeric channel is sufficient to engender a strong dominant negative effect over the receptor’s glutamate potency, and that these channels are not as drastically affected by the variant as in the cases of the pure GluN2B-di-heteromeric channel containing two GluN2B variants (Table 2). Of note, these observations are in support of our previous demonstration in which we have also observed slightly improved EC_50_ values when we co-injected mixtures of mRNA of both *wt-* and variant-subunits (to try favoring the formation of mixed di-heteromers) (see [17]). However, previous attempt necessarily yielded a mixture of multiple channel subtypes in a single cell (i.e., GluN2B-GluN2B, GluN2B-GluN2B*, GluN2B*-GluN2B*) from which we could not draw firm conclusions. These results demonstrate that glutamate potency is not exclusively governed by the least affine subunit, as previously suggested [19, 21, 33, 39]. Instead, EC_50_ values are adjusted (though not equally) by both glutamate binding subunits (*wt* and variant). Our results also provides an elegant demonstration on the necessity of the *liganding* of all subunits for channel opening, strongly supporting previous observations [40–42]. Together, not all GluN2B-di-heteromers are as negatively affected by even the most drastic LoF variants to be described for *GRIN2B*. These may be explained, at least partially, by potential cooperativity between receptor subunits (e.g., [43–45]), but possibly, also due to other, and less affected features in mixed GluN2B-diheteremers (below).

**Fig. 3:**
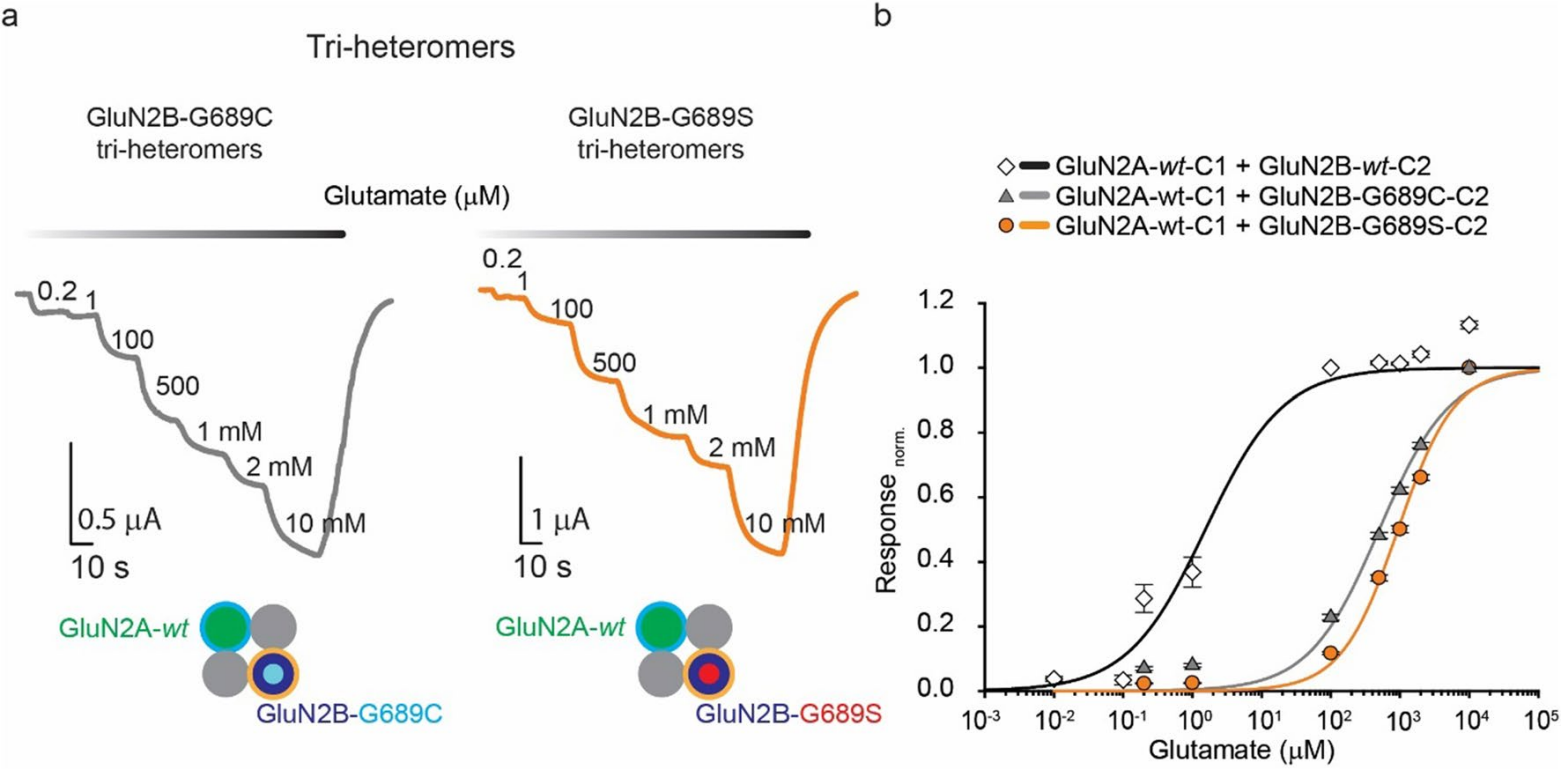
GluN2B-variants reduce glutamate potency of tri-heteromeric receptors. **a** Representative traces from oocytes expressing tri–heteromers composed of GluN1-1a-*wt* with GluN2A-*wt*-C1 (green filled circle with cyan outline in bottom cartoon) and GluN2B-G689C-C2 (dark blue circle with cyan filled circle and orange outline in bottom cartoon) or with GluN2B-G689S-C2 (dark blue circle with red filled circle and orange outline), depicted in grey and orange traces, respectively. Traces show responses to increasing glutamate concentrations. Glutamate (and glycine) application is noted by white-to-black gradient bar above traces. Glutamate concentrations (in μM) are noted next to current steps. Concentrations in mM are explicitly noted next to steps. **b** Summary of dose–response curves for tri–hetereomeric GluN2A/GluN2B-receptors, colored-coded as in (**a**) and (Supplemetary. 1a). Data points represent mean ± SEM. Dose response curves were generated by fitting the normalized data using the adapted Hill equation (see Methods)

We next examined the effect of the variants over the more common, if not the dominant, channel-subtype in the adult brain, namely tri-heteromeric channels composed of GluN2A and GluN2B-subunits [7]. GluN2A-*wt* tagged with the C1 tail, co-expressed with C2-tagged GluN2B variants, also exhibited a dramatic decrease in glutamate potency, yielding ~ 300 and ~ 600-fold increases in EC_50_ by the GluN2B-G689C and GluN2B-G689S variants, respectively, compared to GluN2A-*wt* + GluN2B-*wt* tri-heteromeric channels (Fig. 3**, **Supplementary 1a, b and Table 2). In these instances, we also detected very minimal leak currents (~ 3–7%, Supplementary. 1b, c), as previously shown [8]. These render other ‘leak’ channel compositions less significant in affecting (i.e., adjusting) the observed glutamate potencies (EC_50_).

### Mixed di- and tri-heteromeric channels containing the GluN2B-variants respond differently to GluN2B-selective potentiators

**Fig. 4:**
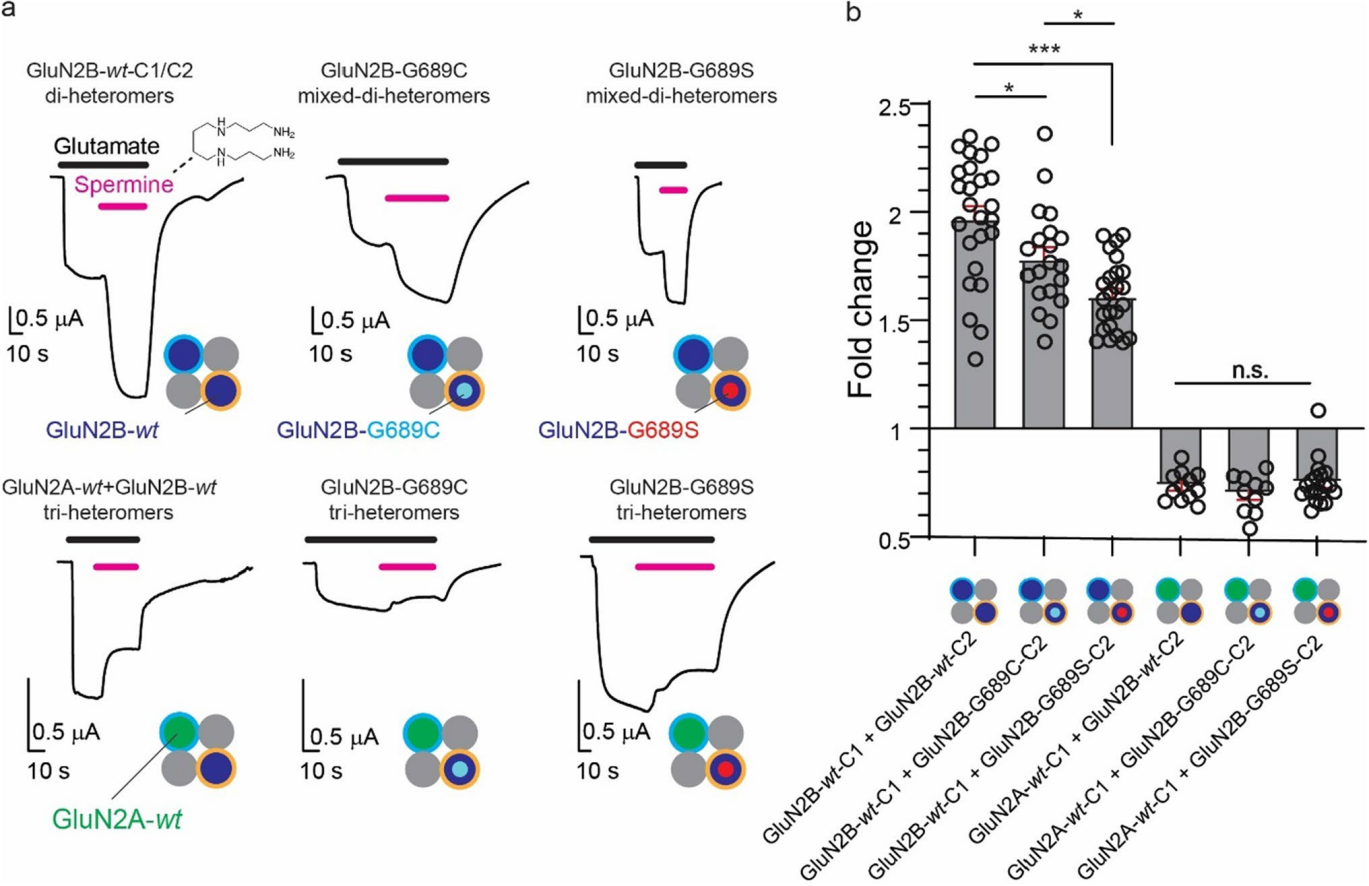
Spermine potentiates only *mixed* di-heteromeric receptors containing two GluN2B subunits. **a** Representative traces from oocytes expressing pure or *mixed* di-heteromers containing the GluN1-1a-*wt* subunit co-expressed with GluN2B-*wt*-C1 and GluN2B-*wt*-C2 or GluNB-G689C-C2 or GluN2B-G689S-C2 (top; left, middle and right traces, respectively) or tri-heteromers assembled from GluN1-1a-*wt* and GluN2A-*wt-*C1 (green) with GluN2B-*wt*-C2 (bottom left) or GluN2B-G689C-C2 (bottom middle) or GluN2B-G689S-C2 (bottom right) in response to 200 μM spermine (pink bars above traces) at pH 7.3, with 5 mM glutamate (and 100 mM glycine) (black bars above traces). Molecular structure of spermine is shown above first panel. **b** Summary of spermine potentiation (fold change) of di- and tri-heteromers. *p < 0.05; ***p < 0.001; *n.s.* not significant. Significance was assessed by one-way ANOVA with Tukey post hoc test

To assess whether channel activity could be rescued pharmacologically, we turned to examine spermine, a GluN2B-selective PAM [46]. Of note, we have previously found that purely di-heteromeric channels, containing two copies of the variant, fail to respond to the drug owing to an unexpected, and severe, reduction in the variants’ pH-sensitivity [17]. We therefore initially examined mixed di-heteromers’ proton-sensitivity and find that the latter display similar responses as their *wt*-counterparts, whereas purely di-heteromers containing the C1/C2-tagged variants exhibited strong and significant reductions, as demonstrated for their non-tagged counterparts (Supplemantary. 2) [17]. We thereby proceeded with testing spermine over the various channels, and indeed observed that mixed di-heteromeric receptors (composed of GluN2B-*wt*-C1 co-assembled with GluN2B-G689C-C2 or GluN2B-G689S-C2) undergo robust, albeit slightly lower, potentiation by spermine compared to *wt* (tagged) receptors (Fig. 4a, b). In these instances, spermine-evoked currents of the mixed di-heteromers were equal or larger than the maximal basal current amplitudes (I_max_, i.e., maximal currents obtained prior addition of spermine) of the *wt* receptors (Suppl. 3a). Interestingly, all GluN2A-*wt-*containing tri-heteromeric receptors were non-responsive to spermine (Fig. 4a). In fact, under our experimental conditions (see Methods), all tri-heteromers underwent slight inhibition by spermine, regardless of the identity of the GluN2B subunit (whether *wt* or variants) (Fig. 4b). The only diverging aspect between the different channels is a missing interface between the GluN1-1a and GluN2B subunits. This delineates that potentiation by spermine minimally requires one GluN2B-subunit with an intact proton sensitivity, but mandates two intact GluN1-1a-*wt*—GluN2B-*wt* interfaces (Table 2) [46]. Thus, although pure di-heteromeric G689C- and G689S-variants retain two intact spermine-binding domains, they lack a single subunit with an intact proton sensitivity [17]. These observations thereby argue against the use of spermine as a possible treatment for GluN2B’s LoF variants, certainly postnatally during which period tri-heteromers are highly abundant. In fact, the use of spermine may worsen the clinical phenotype by inhibiting tri-heteromers. On a side note, due to the different effects of spermine over variant incorporated channels, we also tested the potency of an additional compound which binds the interface between the GluN1a and GluN2B subunit, namely we examined ifenprodil inhibition rate, which functions as a GluN2B specific negative allosteric modulator [2, 8]. We did not observe any differences between the different groups (Supplemantary. Figure 4).

**Fig. 5:**
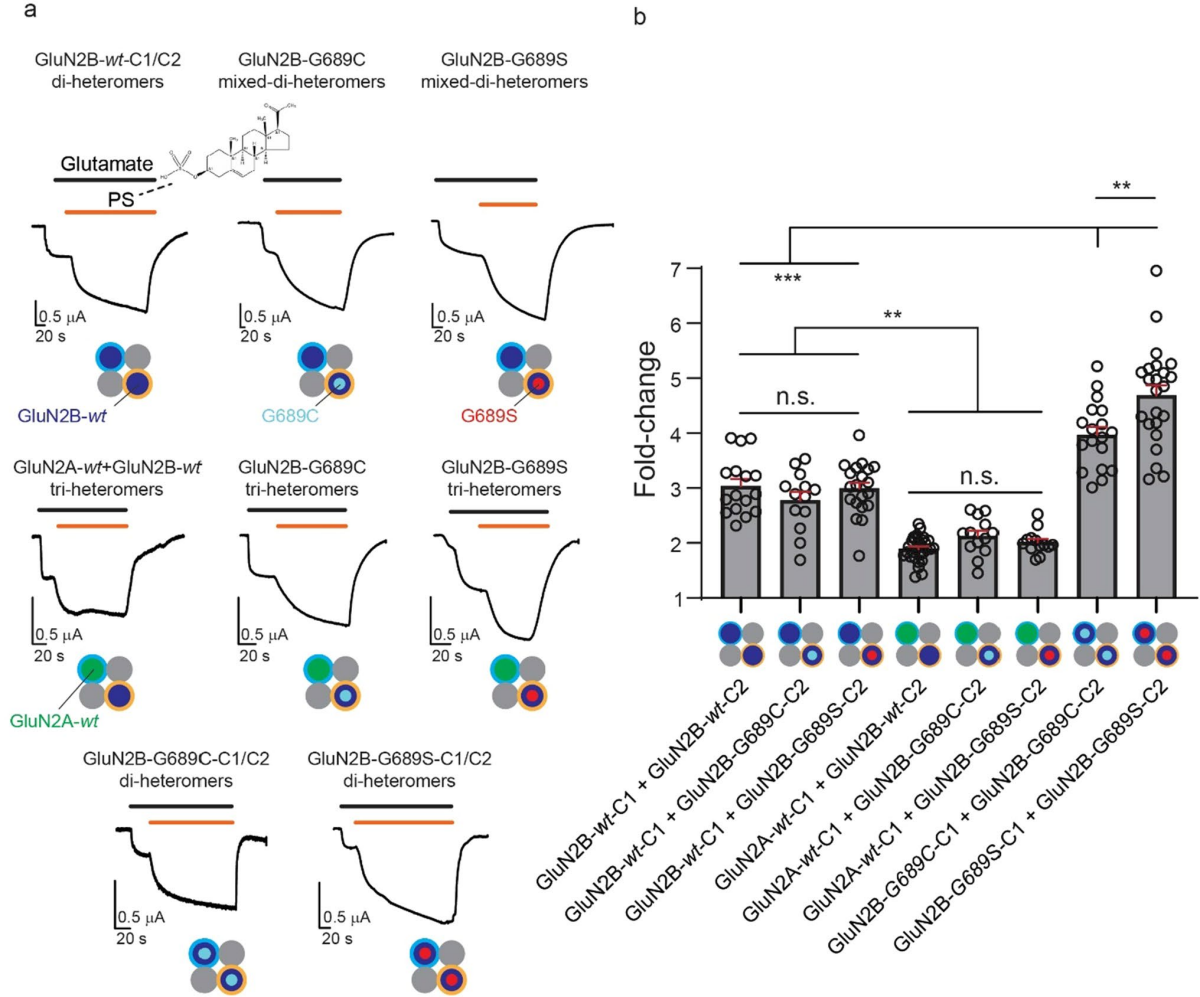
PS effectively potentiates all channel types regardless of subunit composition and GluN2B-variants. **a** Representative traces from oocytes expressing pure or *mixed* di- or tri-heteromers in response to 100 μM PS (orange bars above traces) in the presence of 5 mM of glutamate (100 mM glycine) (black bars above traces). Molecular structure of PS is shown above first panel. **b** Summary of PS-potentiation (fold). **p < 0.01; ***p < 0.001; *n.s.* not significant. Significance was assessed by one-way ANOVA with Tukey post hoc test

Next, motivated by our results with spermine, we reverted to explore another well-established, although slightly less specific, PAM of both di-heterometric GluN2A- and GluN2B-containing receptors, explicitly the neurosteroid—PS [47, 48]. Notably, and to the best of our knowledge, the use of PS on tri-heteromeric channels has yet to be explored. Purely *wt* or mixed di-heteromeric receptors containing GluN2B-subunits exhibited similar potentiation (~ threefold increase) by 100 M PS, as well as all tri-heteromeric types (~ twofold increase; whether with a *wt* or GluN2B variants) and, perhaps the most striking, largest potentiation was obtained for purely variant di-heteromers (G689S- ~ fivefold, G689C- ~ fourfold increase) (Fig. 5a, b and Table 2). As with spermine, PS rescued I_max_ by enhancing it to, or past, the mean I_max_ of the control group (prior addition of PS) (Supplemantary. 3b). The slight differences in potentiation between the different groups could not be attributed to the fact that we did not reach steady-state currents following PS application, as application times of PS were, on average, three time the response’s time constants (τ) (Supplemantary. 5a, b), and we observed a very weak relationship (R^2^ = 0.09) between the response to PS and time of its application (Supplemantary. 5c–k). The very slow potentiation of the currents is likely due to a combination of the very large size of the oocyte, slow perfusion system (gravity-based) and the slow mechanism of action of the neurosteroid (partitioning into the membrane [48, 49]), but it may also reflect diverging potentiation mechanisms of PS over GluN2A *vs*. GluN2B subunits (e.g., see [47]). We therefore suggest that, whereas the precise extent of potentiation may be slightly underestimated, our results clearly demonstrate the ability of PS to potentiate all receptor subtypes; rendering this drug particularly pertinent for neuronal cells (below). Lastly, we also examined the effect of olanzapine, a derivative of clozapine, previously suggested to act as a potential ‘enhancer’ of NMDARs [50–52]. Aside the latter, an additional motivation behind the use of olanzapine is the fact that it is an FDA-approved anti-psychotic drug and therefore, if indeed active, presents novel opportunities to quickly obtain approval for treating *GRINopathies* at the clinic. Unfortunately, we find no evidence for any direct effect of three different physiologically relevant concentrations of the drug on di- or tri-heteromeric receptors (Supplemantary. 6a-e), even though it did instigate its established effect (i.e., inhibition) over the human ether-a-go-go (hERG) channels (Supplemantary. 6f) [53].

Together, our results demonstrate that the binding domain of PS is not affected by the LBD mutations in the GluN2B subunits and show anew that PS is a powerful potentiator of tri-heteromers, regardless of whether the receptors include a *wt* or a variant GluN2B subunit, as previously considered for di-heteromers explicitly [29, 48, 54, 55]. These set PS as a potential candidate drug for rescuing, even if only partially, the deleterious effects of the variants over channel currents by enhancing them.

### PS rescues NMDAR-current amplitudes in cultured hippocampal neurons

**Fig. 6:**
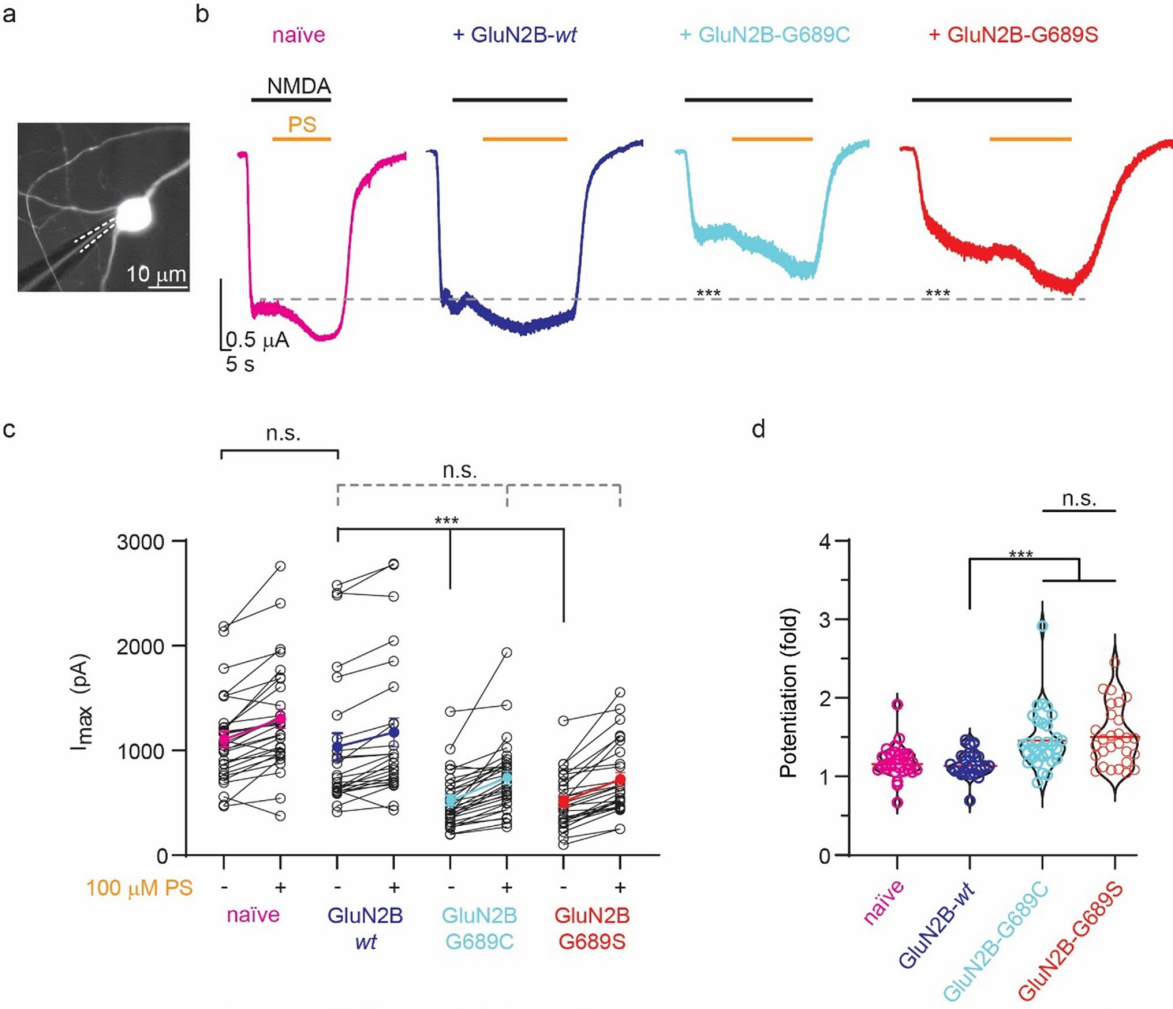
PS rescues NMDAR-current amplitudes in hippocampal neurons overexpressing GluN2B-variants. **a** Representative microscope image of a hippocampal neuron overexpressing a GluN2B-variant and YFP (for visualization); recording pipette is highlighted by dashed white lines. **b.** Whole cell recordings of NMDAR-dependent currents from naïve (pink trace), or overexpressing GluN2B-*wt* (dark blue trace), GluN2B-G689C- (cyan) or GluN2B-G689S (red) in response to 100 μM NMDA (50 μM glycine) (black bars above traces). Application of 100 μM PS is shown by orange bars above traces. Note the strong and significant reduction in the maximal steady-state NMDAR-dependent currents between control and GluN2B variant-expressing neurons (dashed grey line and asterisks; significance taken from (**c**)). **c** Summary of the maximal currents (I_max_), before and after application of PS. Each bullet represents an individual neuron, means (and SEM) are depicted by pink (naive), blue (GluN2B-*wt*), cyan (GluN2B-G689C) or red (GluN2B-G689S).** d** Summary of potentiation (fold change) by PS. ***p < 0.001; n.s., not significant. Significance was assessed by Kruskal–Wallis test followed by Dunn’s multiple comparison post hoc. For (c-d), naïve, N = 4, n = 30; GluN2B-*wt*, N = 3, n = 25; GluN2B-G689C, N = 4, n = 31; GluN2B-G689S, N = 4, n = 28

We quickly turned to examine the functional outcome of the use of PS in neurons. We co-transfected cultured hippocampal neurons with the GluN2B-subunit (i.e., *wt*, G689C, G689S) along a soluble cell marker (eYFP, Methods); relying on endogenous subunits to assemble and traffic the GluN2B-subunits to the membrane (Fig. 6a) [17, 56]. The sole transfection of the GluN2B-subunit (without co-expression of the GluN1-1a-*wt*) is not expected to robustly increase the surface expression of NMDARs and is thereby accepted to be more physiological (see below) [11, 56–60]. Of note, co-transfection of two independent plasmids is commonly done in the field, as it provides high co-transfection efficiencies (> 80%) [61, 62]. To overcome the more serious caveat in co-transfection, namely the potential competition between the two independent DNAs over the transcription machinery (which may yield different levels of expression between the two), we transfected our neuronal cultures with excessively larger concentrations (~ 6-folds) of the plasmid encoding for *GRIN2Bs* than that of YFP in order to favor robust expression of the former [63]. To firstly ensure that YFP-positive neurons were indeed expressing the different GluN2Bs (*wt*, or variants), we measured NMDA-dependent miniature EPSCs (mEPSC_NMDA_) (Methods). Briefly, expression of the variants almost completely eliminates mEPSC_NMDA_s’ frequency [17]. We recapitulated these results by noting a very strong reduction in mEPSC_NMDA_s’ frequency specifically in YFP-positive neurons co-transfected with DNAs for the GluN2B-G689C or GluN2B-G689S variants, whereas naïve or GluN2B-*wt* co-transfected neurons exhibited comparable frequencies (Supplemantary. 7a, b). Overexpression of the GluN2B-*wt* significantly reduced the variability between the cells in the same group. These strongly suggest the expression of the different clones in the YFP-positive cells.

To then try to examine the potential effect of PS over synaptic activity, we perfused neurons with 100 μM of PS (without addition of the ligands, i.e., NMDA). Application of PS enhanced the overall NMDAR-current, as well as significantly increased tonic NMDAR-activity (assessed by addition of AP5) (Supplemantary*.* 7c, compare I, iii then iv) [64]. These robust increases in activity prevented reliable detection and quantitation of single mEPSC_NMDA_ (Supplemantary. 7c, ii), even when both drugs were washed-out (owing to the slow mechanism of PS, potentiation remained evident for several minutes after wash-out of the drug) (Supplemantary. 7c, iv). Nevertheless, the peak amplitude of the total NMDAR-response to PS was comparable between the different groups (Supplemantary. 7c, arrowhead), suggesting that all groups had an equal amount of readily available PS-responsive receptors (Supplemantary. 7d). Therefore, we considered that a more effective approach to discern the positive impact of PS would be to evaluate the potential restoration (rescue) of the maximal current in neurons overexpressing the variants. To address this, we applied 100 μM of NMDA (and glycine) to measure I_max_ in neurons. We selected this concentration (as opposed to using mM concentrations) as it better reflects physiologically-relevant concentrations of glutamate at the synaptic cleft [65, 66], it does not lead to a very rapid decline in neuronal viability and death of the culture, and allows to measure activity from many cells in the same coverslip, without inducing glutamate-excitotoxicity (several minutes of exposure of > 20 μM of NMDA are typically employed to mimic excitotoxicity) [67]. Under these conditions, we assume reduced activation of pure variant-containing subtypes (see Figs. 1, 2, 3 and Table 2). These settings are warranted as they are likely to better represent the in vivo environment in patients as most *GRINopathies* are heterozygous in nature. We first noted that overexpression of the GluN2B-*wt* subunit alone yielded I_max_ amplitudes on par with those of naïve neurons (Fig. 6b, magenta and dark blue traces, c), supporting the notion that overexpression of the GluN2B subunit alone does not increase the pool of NMDARs at the membrane (see above). In sharp contrast, overexpression of the variants engendered a significant reduction in I_max_, suggesting a reduction in the total amount of *wt* receptors at the membranes of neurons (Fig. 6b, cyan and red traces, summarized in c, also see more examples in Supplementary. 7e) [17]. Application of 100 μM PS increased I_max_ of neurons from all groups (Fig. 6c, + 100 μM PS), however the extent of potentiation (fold) by PS was significantly larger in neurons overexpressing the variants (Fig. 6d). These observations are consistent with our observations in oocytes (see Fig. 5b). Importantly, the potentiated currents in neurons overexpressing the variants enhanced the currents to amplitudes reaching the I_max_ of control (naïve or GluN2B-*wt* expressing) neurons (Fig. 6b, dashed grey line and c, grey dashed statistics). These emphasize the ability to rescue the diminished NMDAR-current amplitude by PS and highlight its’ potential as a candidate drug for LoF *GRIN2B* variants.

Next, to examine whether the overexpression of the variants had any effect over the GluN2B-containing receptor pools, we applied 2.5 μM ifenprodil (a selective GluN2B-inhibitor [8, 36]) (Supplementary 7e) which we have previously demonstrated its ability to inhibit GluN2B-*wt* and GluN2B-variant incorporated receptors in a similar manner (Supplementary. 4). At this concentration, the majority (~ 85%) of GluN2B di-heteromers are expected to be inhibited by the drug, with < 40% inhibition of tri-heteromers (GluN2A-containing di-heteromers are less affected by the drug; < 10%) [7]. We obtained a similar inhibition (%) of I_max_ by ifenprodil in all groups, suggesting the overexpression of the GluN2B subunit alone did not engender a robust increase in surface GluN2B-containing NMDARs and that the variants are equally distributed between ifenprodil-sensitive di- and tri-heteromers (Supplementary. 7f). Lastly, to examine the distribution of the variants between synaptic- and extrasynaptic loci, we utilized a standard procedure involving the activity-dependent blocker MK-801 (Methods) [68]. We have previously shown that MK-801 efficiently and potently blocks both variant receptors (and both variants exhibit similar apparent open probability) [17, 69], thereby enabling the use of the drug. Briefly, neurons were continuously bathed in TTX during which they were probed for NMDAR I_max_, which relies on the joint activation of synaptic and extrasynaptic receptors, using 100 μM NMDA (to refrain from activating other GluRs). Subsequently, we washed the 100 μM NMDA and applied MK-801 for 10 min. During this 10-min phase only synaptic receptors, which will open following action-potential independent miniature neurotransmission (minis), will be blocked by the activity-dependent blocker MK-801. Therefore, upon a second application of 100 μM NMDA the fraction of extrasynaptic NMDAR is exposed (Supplementary 8a, inset) [68] as synaptic receptors are blocked. In neurons overexpressing the GluN2B-*wt* subunit, we obtained an extrasynaptic fraction of 38%, highly consistent with previous reports [68, 70]. Concurrently, overexpression of the variants, yielded a significantly and higher fraction (~ 50%) of remaining current (Supplementary. 8b, c). However, owing to fact that the overexpressed variants have significantly reduced potency (see Figs. 1, 2, 3 and Table 2) and cause severe LoF, the results obtained from this experiment do not accurately reflect a larger pool of extrasynaptic receptors (in the case of variant overexpressing cells), but rather shed light on the distribution (synaptic vs extrasynaptic) of *wt—*receptors for the following reasons. Firstly, 100 μM NMDA does not saturate variant receptors (whether pure, mixed di- or tri-heteromers, see Table 2), therefore their affect over I_max_ can’t be tested during both applications of NMDA. Secondly, quantal neurotransmission does not open synaptic receptors containing variant GluN2B-subunits, attested by the strong reduction in the frequency of mEPSC_NMDA_ (see Supplementary 7a, b). Thus, during the 10-min phase of MK-801 application, solely *wt*-receptors are blocked by spontaneous synaptic activity. Accordingly, during the second bout of 100 μM NMDA, solely the remaining *wt*-receptors at the extrasynapse are activated and exposed. Consequently, in neurons overexpressing the variants, the observed increase in the fraction of extrasynaptic receptors, specifically, implies that the number of *wt*-receptors is necessarily decreased from the synapse most likely due to the incorporation of the variants into receptors at the synapse. Moreover, the strong reduction in mEPSC_NMDAR_ frequency strongly supports the notion of the removal of *wt*-receptors from the synapse, as opposed to the direct increase in the number of *wt*-receptors are the extrasynapse (see Supplementary 7a).

## Discussion

The formation of tri-heteromeric receptors, consisting of two GluN1 subunits and two different GluN2 subunits, notably GluN2A and GluN2B, is becoming increasingly recognized in the field [1, 7, 71]. Additionally, it is gaining wide acceptance that *GRIN* genes variants give rise to a diverse range of encephalopathies and conditions such as epilepsies, and intellectual disabilities, especially observed in pediatric patients [72]. However, despite major advancements in each matter, our understanding of how *GRIN* variants specifically impact the function of tri-heteromeric receptors, particularly at the synapse, and sensitivities to pharmacology, remains limited (i.e. [10, 21, 23, 73, 74],). Thus, better understanding of the receptors’ function and pharmacology may help accelerate drug discovery.

Here, we try to bridge this gap for two extreme GluN2B variants, specifically GluN2B-G689C and GluN2B-G689S [17], in the context of pure and mixed di- and tri-heteromers. We first focused on the most detrimental feature of both analogous variants over receptor function, namely their ultrapotent (~ 2000-fold) reduction in glutamate potency. These GluN2B variants include a mutated residue (p.G689) at the opening of the LBD, which likely influences the correct coordination and binding of glutamate; translating to extremely high EC_50_ values (~ mM) [17]. Here, we show anew that a single GluN2B-subunit, whether assembled with a GluN2B-*wt* subunit to form a *mixed* di-heteromer or with a GluN2A-*wt* subunit within a tri-heteromer, strongly reduces glutamate potency of both receptor types. Nevertheless, the ensuing potencies are less extreme than those obtained from purely di-heteromeric receptors containing two GluN2B-variants (Figs. 1, 2, 3; Table 2). On the one hand, this observation was somewhat surprising as NMDARs opening requires all four subunits to be *liganded*, implying that the least affine subunit should limit (i.e., govern) the glutamate potency of the entire receptor complex with an EC_50_ equal to that of the least affine subunit [19, 21, 33, 39]. However, our results are consistent with several reports showing that mixed di-heteromeric receptors exhibit intermediate functional properties [10, 19, 23, 32, 39], including improved glutamate affinity when specifically addressing LoF variants [21, 33]. Explicitly, we explored the literature and found multiple reports in which mixed di- and tri-heteromers, containing different *GRIN* variants, have been compared side-by-side with the pure di- and tri-heteromers counterparts (18 *GRIN* variants in total, Table 1). The data (including our data from this report) reflect 6 GluN2A-GoF, 3 GluN2A-LoF, 4 GluN2B-GoF and 5 GluN2B-LoF variants that have been explored within various NMDAR compositions. We examined the relationship between the glutamate potency (EC_50_) of the least affine heteromeric receptor (whether purely *wt* or variant-containing receptors, depending on whether the mutation is GoF or LoF) with the corresponding mixed di-heteromers containing only one variant (Supplementary 9a) and discovered that mixed receptors containing one variant exhibit a ~ fourfold (on average) increase in apparent affinity compared to the least affine receptor (Supplementary 9b,c; see Table 1 for details), thus displaying an intermediate effect. This glutamate potency ratio may help to predict glutamate potency of mixed variant receptors based on the characterization of their pure variant counterparts alone, minimizing the need for excessive experimentation. Collectively, these observations suggest that the amelioration in glutamate potency of mixed di-heterodimers could stem from positive cooperativity between the subunits (e.g., [43–45]) (but see Hill coefficients in Table 2). Regardless the exact mechanism, the negative effect of the two variants over mixed di- and tri-heteromeric receptors is sufficiently strong so that their improved EC_50_’s are still below the suggested concentrations of glutamate at the synaptic cleft [65, 75, 76], rendering pure di-heteromers, but also mixed di- and tri-heteromers, oblivious (i.e., silent) to neurotransmission (Supplementary. 7a, b) [17, 77, 78]. This very strong reduction in synaptic activity by the overexpression of the variants suggests that there are very few remaining *wt*-receptors at the synapse, instead these are replaced by di- and/or tri-heteromeric receptors containing the variants (Supplementary. 7b). This replacement is coupled to a strong decrease in I_max_ in neurons overexpressing the variants compared to naïve or GluN2B-*wt* expressing neurons (Figs. 6b, c and additional examples in Supplementary 7e and. 8a), and a supposed increase in the fraction of extrasynaptic receptors necessarily containing only *wt* subunits (Supplementary. 8c). These dominant negative effects diverge from haploinsufficient *GRINopathies,* that may also result in reduction in the expression levels (and I_max_) of NMDARs (e.g., by de novo early stop codons), and are suggested to lead to more severe phenotypes in patients [28, 35, 74, 79–81]. For instance, patients with *GRIN* truncations (i.e., haploinsufficiency) present milder intellectual disabilities [25].

The diverse array of channel types resulting from the combination of the two NMDAR variants with the different GluN2-subunits (GluN2B-*wt* and GluN2A-*wt*) has prompted us to investigate their responsiveness to established and selective GluN2B reagents. First, we find that spermine remains a strong potentiator of mixed di-heteromers containing the GluN2B-variants (Fig. 4), despite our previous observations that purely variant di-heteromers are poorly responsive (or even inhibited in the case of the G689S variant) to spermine. The positive responses of mixed di-heteromers, compared to the null effect over pure variant di-heteromers results from the restored pH-sensitivity of mixed di-heteromers (Supplementary. 2). This was surprising as the minimal and essential rules of engagement for potentiation by spermine are not well established, particularly in the case of tri-heteromers [7, 46]. Here, we incidentally define the latter by demonstrating that spermine potentiation minimally depends on a single GluN2B-subunit with intact proton sensitivity but requires two intact interfaces between GluN-1a-*wt* and GluN2B-*wt* subunits (Table 2). Indeed, all tri-heteromers examined did not respond to spermine, rather undergo modest inhibition (0.75-fold) by the drug (Fig. 4b) (in agreement with [82]). A plausible explanation for these observations is that the transduction of the effect of spermine requires coordinated action between all GluN1-GluN2B dimers, rendering the dimer-of-dimer arrangement of the receptor essential, in which case tri-heteromers are missing an essential interface. Thus, despite the positive effect of spermine over mixed di-heteromers (which should be prevalent in prenatal stages), its use should be avoided postnatally as a large fraction of receptors at synapses are likely tri-heteromers [7, 9] and these are readily nonresponsive to the drug (Fig. 4b).

Given the ineffectiveness of spermine, we evaluated the potential of PS, a potentiator belonging to the neurosteroids family. Intriguingly, and to the best of our knowledge, the impact of PS over tri-heteromers has not been previously investigated. Therefore, we were unable to anticipate the extent of its potency (Fig. 5). Interestingly, our findings reveal that PS exhibits considerable efficacy in potentiating all tested receptor types, including mixed di- and tri-heteromers, even though its mechanism of potentiation is pH-dependent [83] and the variants exhibit diminished pH-sensitivity [17]. In fact, the highest level of potentiation by PS was observed for purely di-heteromeric receptors composed of two copies of the variants within a single receptor (Fig. 5b). These are supported by larger potentiation of NMDAR-currents in primary cultured neurons overexpressing the GluN2B variants (Fig. 6d). A plausible reason for the stronger PS potentiation of the purely variant di-heteromeric receptors is the fact that high affinity receptor types (e.g., *wt* receptors) exhibit responses that decline fast upon agonists application, in a mechanism described as “dis-use dependent” potentiation[47, 84, 85]. According to this mechanism, PS induces an increase in glutamate affinity so that it also leads to its rapid dissociation in the presence of high glutamate concentrations, resulting in overall reduced potentiation. Thus, receptors with lower glutamate potency (e.g., purely variant receptors) should exhibit more prolonged effects by PS, represented by higher extents of potentiation, as in the case of purely variant di-heteromeric receptors (Fig. 5b). This notion is also somewhat in-line with our observations with the mixed di- and tri-heteromers (with less reduced glutamate potency than purely variant di-heteromeric receptors) that are less potentiated by PS in comparison to purely variant-containing di-heteromeric receptors. However, this hypothesis fails to explain why *wt* receptors and mixed di or tri-heteromers bearing a single GluN2B-variant are equally potentiated by PS (Fig. 5). Another interesting observation is the differing (and larger) extent of potentiation observed in oocytes compared to hippocampal neurons. Possible explanations for this disparity may arise from differences in the rates of potentiation by PS in these two preparations, which could be influenced by the robust Ca^2+^-dependent inhibition of NMDARs, primarily observed in neurons but not oocytes [86]. Additionally, the expression of additional GluN2-subunits in neurons, particularly those undergoing reduced potentiation (GluN2A [47], see also Fig. 5b) or inhibition (GluN2D [47]) by the drug, may contribute to these differences. Taken together, our results show that the presence of the GluN2B variants does not impair the responses of different channel subtypes to PS, instead it can even ameliorate the responses of purely variant di-heteromeric receptors. Despite the positive effects of PS on NMDAR channel activity, it is important to acknowledge that PS lacks specificity in its action. Previous studies have reported PS to act as a negative allosteric modulator (NAM) of GluN2C- and GluN2D-containing di-heteromers [87] (although not tested on tri-heteromers containing these subunits), as well as of the GABA_A_ receptor [47]. These may have serious negative outcomes, for instance inhibition of the GABA_A_ receptors may potentially contribute to increased susceptibility to epileptic seizures. Therefore, the therapeutic potential of PS for *GRINopathies* requires further scrutiny, however it sheds light on the potential of using neurosteroids as a treatment for the disease. Fortunately, there are several alternative neurosteroids, such as 24(S)-hydroxycholesterol (24(S)-HC), a major cholesterol metabolite in the brain [55]. This steroid can potentiate all NMDAR-subtypes alike, with no known effect over GABA_A_ receptors, and has several synthetic analogues (e.g., SGE-201, SGE-301) [55]. The latter are particularly of interest, as they are suggested to exhibit higher stability and can reach higher effective concentrations in the blood. Additionally, as each steroid exhibits slightly different effects and preferences towards various subtypes, we suggest a more complex therapy involving a combination of neurosteroids to obtain synergistic effects and, perhaps, even higher selectivity owing to the fact that different steroids act on alternative sites on the subunits [54]. Conversely, we propose a novel approach for elevating 24(S)-HC in the brain of GRIN *patients* through the stimulation of cytochrome P450 46A1 (CYP46A1) activity. CYP46A1 is responsible for the conversion of cholesterol to 24(S)-HC; which is a byproduct of the FDA-approved drug Efavirenz (an unrelated anti-retroviral compound [88]). This treatment has not been explored with regard to *GRINopathies* by others. However, we could not have explored the Efavirenz treatment owing to challenges encountered in generating transgenic animals expressing the *GRIN2B*-G689C/S variants (see Supplementary Text).

In summary, our study focused on assessing the impact of a single variant on the function of di- and tri-heteromeric NMDARs. Our findings reveal that whereas a single dysfunctional subunit exerts a dominant negative effect on glutamate potency, it does not solely determine the impact on allosteric properties of the receptor, such as spermine and PS potentiation. This positive observation supports the idea that neurosteroids may be of use in the case of *GRINopathies*, and hopefully will help pave the way towards assessing new therapeutic approaches as suggested above. Our results also underscore the necessity to investigate how different variants affect various receptor subtypes as conclusions drawn from observations solely on di-heteromeric receptors may not fully capture the complexity of the system nor reflect the outcome on other receptor types. Overall, our study contributes to the ongoing efforts in understanding the underlying pathophysiology of *GRINopathies* and provides valuable insights for the development of potential treatments.

## Methods

### *Xenopus Leavis* oocytes extraction

*Xenopus Leavis* oocytes were collected, processed, and injected with mRNA, as previously described [17, 89]. Briefly, female frogs were anesthetized, and their ovaries were harvested. Ovaries were then treated with collagenase in ND96 Ca^+2^-free solution (in mM: 96 NaCl, 2 KCL, 1 MgCl2, 5 HEPES, pH = 7.4) for 20 min at RT, to isolate and defolliculate the oocytes. Subsequently, oocytes were washed with ND96 Ca^+2^-free solution and stored in enriched ND96 medium (NDE) consisting of ND96 added with 2.5 mM sodium pyruvate, 1.8 mM CaCl_2_, 100 mg/ml streptomycin, and 62.75 mg/ml penicillin. Lastly, hand-picked stage V oocytes were identified, separated, and incubated overnight at 18 °C, and then injected with mRNA.

### Dissociation, culturing, maintenance, and transfection of primary hippocampal neurons

Cultures of hippocampal primary neurons were established as previously stated [90]. Briefly, extracted rat neonates (P0) hippocampi were dissociated and plated onto 12 mm poly-D-lysine (Sigma-Aldrich, Cat. #P6407)- treated glass coverslips. Cultures were then maintained in an enriched growth media and grown at 37 °C and 5% CO_2_. Following five days in-vitro (DIV), growth medium was supplemented with 4 μM cytosine-arabinoside (ARA-C) to suppress glia proliferation. At nine DIV, neurons were co-transfected using the calcium-phosphate method with 2 different plasmids: 0.3 μg DNA of eYFP and 2 μg GluN2B-*wt* or GluN2B-G689C or GluN2B-G689S. To avoid recordings from cells transfected only with the fluorescent reporter, the ratio between the different plasmids during co-transfection favored the plasmids encoding GluN2B-*wt*/G689C/G689S. Recordings were performed four to seven days past transfection.

### Molecular biology and in vitro mRNA preparation

Rat GluN1-1a-*wt*, rat GluN2A-*wt*-C1/2 and rat GluN2B-*wt*-C1/2 plasmids were obtained from.

Prof. Hansen K.B. (Montana University). Rat GluN2B-G689C-C1/2 and Rat GluN2B-G689S-C1/2 were generated using the QuikChange Site-Directed Mutagenesis Kit (Agilent,Cat. # 200,518). Primers for GluN2B-G689C Mutagenesis: sense- 5′-CGCTTTGGGACTGTGCCCAATTGCAGCACAGAGAGGAATATCCG -3′, antisense- 5′-CGGATATTCCTCTCTGTGCTGCAATTGGGCACAGTCCCAAAGCG-3′; for GluN2B-G689S: sense- 5′-CCGCTTTGGGACCGTGCCCAACAGCAGCACAGAGAGAAATATCCG-3′, antisense- 5′-CGGATATTTCTCTCTGTGCTGCTGTTGGGCACGGTCCCAAAGCGG-3′. All PCR products were fully sequenced. For *in-vitro* mRNA transcription, plasmids were linearized with NotI, and transcription was obtained by mMessage-mMachine T7 kit (Thermo Scientific, Cat. #AM1344). Subsequently, mRNA concentrations were measured using a spectrophotometer. For selective expression of different compositions of NMDARs, mRNA of GluN1-1a was co-injected with GluN2A/B with different tails (C1 or C2) at a ratio of 1:3.75:3.75 respectively, and in total ~ 28 ng mRNA/oocyte. For assessment of leak expression, mRNA of rat GluN1-1a was co-injected with GluN2A-wt-C1 or GluN2B-wt-C1 (or both) at similar ratios and amounts as described above. Recordings were then performed 24–72 h after injection. For hERG expression, 25 ng mRNA/oocyte was injected. Lastly, in experiments involving non-tagged NMDAR, mRNA of GluN1-1a and GluN2B (or GluN2A) were co- injected at a ratio of 1:7.5 respectively, and in total ~ 28 ng mRNA/oocyte.

### Selective cell-surface expression of NMDARs in oocytes

Selective expression of recombinant GluN1-1a/GluN2A/GluN2B tri-heteromers was performed utilizing a method previously described [8]. Briefly, GluN2B and GluN2A-subunits were tagged at their carboxy-termini (CT) with unique ‘tails’, denoted C1 and C2. Tails consist of linkers including leucine zipper motifs and an ER retention signal from GABAB_1_ and GABAB_2_ followed by an additional di-lysine KKXX ER retention/retrieval motifs. The CTD of the GluN2B subunit was replaced by the distal CTD of GluN2A subunits that contained the C1 or C2 tags, namely residues past 844 amino acids in the GluN2B subunit were replaced by residues 844–1541 and 844–1533 from the GluN2A clones that had C1 and C2 tags inserted, respectively. Selective cell-surface expression is then achieved only when C1-tailed subunit interacts with a C2-tailed subunit to form a coiled coil structure between the tails, thus masking the ER retention signals, enabling trafficking of the dimer (along two GluN1 subunits) to the surface. Receptors assembled from C1-tailed subunits or from C2-tailed subunits remain in the ER.

### Two electrode voltage clamp recordings in *Xenopus* Laevis oocytes

Two electrode voltage clamp (TEVC) recordings were carried out 24–72 h after mRNA injections, as previously described [17, 37]. We use a commercial amplifier (Warner Instruments, USA) and Digitizer (Digidata-1550B; Molecular Devices, USA), controlled by the pClamp10 software (Molecular Devices, USA). Electrode were made by pulling glass capillaries (Narishige, Japan) filled with 3 M KCl, into which we inserted chlorinated silver wires. Stage V oocytes were then clamped at (− 60) mV and perfused with Barth solution (in mM): 100 NaCl, 0.3 BaCl_2_, 5 HEPES, at pH = 7.3 (adjusted by KOH, ~ 2.5 mM) using VC3-8xP gravity flow perfusion system (ALA scientific instruments). Glutamate dose response experiments were performed with glutamate concentrations ranging between 0.2 μM up to 10 mM (all containing 100 μM glycine); higher concentrations then 10 mM glutamate were avoided to avoid non-specific currents. To assess sensitivity to pH, oocytes were perfused with Barth solution at varying pH’s, ranging from 6.0 to 8.3, supplemented with 5 mM glutamate and 100 μM glycine. For hERG channel recordings, oocytes were clamped at (− 60) mV for 1 s, followed by a voltage jump to 20 mV for 4 s, and an additional voltage jump to (− 50) mV for 6 s and return to baseline voltage (see Supplementary. 6f), as previously described [53].

### Electrophysiology (whole cell patch clamp) of primary rat cultured neurons

We patched YFP-positive neurons at 13–18 DIV. YFP was excited by X-Cite LED illuminator (Excelitas Technologies). Electrode were made by pulling glass capillaries to resistance of 5–10 MΩ. Electrodes were filled with an intracellular solution containing (in mM): 135 K-gluconate, 10 NaCl, 10 HEPES, 2 MgCl_2_, 2 Mg^2+^-ATP, 1 EGTA, pH adjusted to 7.3 with KOH. Patch-clamp recordings were performed with Axon MultiClamp 700B amplifier and Axon Digidata 1440A acquisition system. In all experiments, recordings were performed using a 10 kHz sampling rate and low pass filter. Neurons were clamped at (-70) mV. Recordings of NMDAR dependent mEPSC_NMDA_ were performed by perfusing the neurons with an extracellular solution containing (in mM): 138 NaCl, 10 Glucose, 5 HEPES, 2.5 CaCl_2_, 1.5 KCl, pH = 7.4 (adjusted by NaOH), and (in μM): 50 glycine and 20 CNQX (Alomone labs, Cat #: C-140), 10 gabazine (Alomone labs, Cat #: G-215), and 1 TTX (Alomone labs, Cat #: T-550) using VC3-8xP gravity flow perfusion system (ALA scientific instruments). Cells were recorded for ~ 3 min. For assessing the total NMDAR-dependent current (I_max_), neurons were then perfused with extracellular solution (see above) supplemented with 100 μM of NMDA (Alomone labs, Cat #: N-170) for exclusive activation of NMDARs. Potentiation was assessed by perfusing cells with the extracellular solution along with 100 μM NMDA and 100 μM of PS (Cat. #P162) in 0.2% DMSO. Lastly, Inhibition of GluN2B-containing receptors was evaluated by application of 2.5 μM ifenprodil (Alomone labs, Cat #: I-105). Cells that didn’t reach steady-state after application of 100 μM NMDA were excluded from analysis. Extent of potentiation by PS was evaluated at steady-state.

Evaluation of the fraction of extrasynaptic NMDARs was done as previously described [68]. Briefly, neurons were initially perfused with extracellular solution (see above) supplemented with 100 μM of NMDA to assess the total (synaptic and extrasynaptic) NMDAR-dependent current. Subsequently, the 100 μM of NMDA was thoroughly washed for 2 min (to refrain from activation of NMDAR by remaining NMDA), then neurons were perfused with the extracellular solution supplemented with 1 μM MK-801 for 10 min to exclusively block synaptic receptors (which are activated by action-potential independent miniature neurotransmission). Extrasynaptic NMDAR-dependent currents were then measured by washing the cells from excess MK-801 for 2 min followed by a second application of 100 μM NMDA. Evaluation of the fraction of the extrasynaptic NMDARs was done by dividing the maximal current remaining after MK-801 application by the current obtained before the MK-801 treatment.

### Potentiation of NMDAR-currents by drugs

Spermine (Cat. #S3256), PS (Cat. #P162) and olanzapine (Cat. #O1141) were purchased from Sigma-Aldrich. Spermine stock solution (200 mM, in water) was freshly made on the day of each experiment. 50 mM and 10 mM stock solutions of PS and olanzapine, respectively, were made in 100% DMSO. To avoid PS degradation, aliquots were kept at -20 °C for up to four weeks and underwent a single thawing process. Aliquots were thawed before each experiment. In *Xenopus Laevis* oocytes, potentiation was evaluated by application of the drugs (200 μM spermine, or 100 μM PS or 0.1–10 μM olanzapine) in the presence of 5 mM glutamate and 100 μM glycine. When assessing potentiation by PS, all other solutions also included equal amounts of the DMSO carrier (0.2%).

### Data and statistical analysis

Electrophysiology data were analyzed by Clampfit 11.2 (Molecular Devices, USA), plotted using GraphPad 8 or SigmaPlot 11(Systat software, Inc.). EC_50_ and IC_50_ values were extracted by fitting the data to adapted Hill equations: Eq. (1) Response = 1/ (1 + [(glutamate)/EC_50_]^nH^)and Eq. (2) Response = minimum + (1–minimum)\ 1 + [(pH)/IC_50_]^nH^), respectively. For Eq. (1) and Eq. (2) (glutamate) indicates glutamate concentration in μM, (pH) indicates (− log) of proton concentration, nH is the Hill slope, EC_50_ is the agonist concentration that induce half of the maximal response and IC_50_ is the inhibitor concertation required to induce half of the maximal response. In all TEVC recordings in oocytes, the responses were normalized to current amplitude obtained from that individual cell. During glutamate dose response experiment the responses of *wt*-receptors, at different glutamate concentrations, were normalized to the response attained 100 μM glutamate, whereas, in the case of variant incorporated NMDAR compositions, the different current amplitudes were normalized to I_max_ obtained at 10 mM glutamate. All data are shown as mean $\pm$ SEM. In all experiments N indicates the number of independent experiments, whereas n indicates the number of cells recorded. In each experiment, data underwent normality test prior to statistical assessment. Normally distributed data were assessed for significance by using paired t-test or one-way ANOVA for multiple group comparison and post hoc Tukey test or Dunnett's test. Similarly, in the case of non-normally distributed data significance was assessed by Mann Whitney test or Kruskal–Wallis ANOVA on ranks test followed by Dunn’s multiple comparison post hoc; n.s., non-significant; *p < 0.05; **p < 0.01; ***p < 0.001. Extent of potentiation (by spermine or PS) or inhibition (by ifenprodil) reflects the current after application of the drugs (in the presence of NMDAR agonists) divided by the steady-state glutamate (or NMDA) current before the treatment. In the case of hERG channels, inhibition by olanzapine was defined as the ratio between peak amplitudes at (− 50) mV after and before the application of the drug. Leak expression was evaluated by normalizing the maximal current (I_max_) of each ER-retained NMDAR composition to I_max_ of control group (which was recorded in all experiments). Estimation of activation time constant (τ) for potentiated currents by PS, was performed by fitting PS- potentiated currents with a standard single-exponential function, from which we extracted the time constant (τ). The ratio in Table 1 was calculated based on the EC_50_ of di-heteromers composed of either *wt* or variants (i.e., *wt*-receptor in the case of GoF variants and pure variant receptor in the case of LoF variant) and the EC_50_ of the mixed di- or tri-heteromeric receptor (composed of a GluN2-*wt* subunit coupled with a GluN2-variant).

### Supplementary Information

Below is the link to the electronic supplementary material.

Supplementary file1 (DOCX 296 KB)

## Data Availability

The authors confirm that the data supporting the findings of this study are available within the article [and/or] its supplementary materials. Raw data that support the findings of this study are available from the corresponding author, [SB], upon reasonable request.
