# Supplementary material for: Rescuing tri-heteromeric NMDA receptor function: the potential of pregnenolone-sulfate in loss-of-function GRIN2B variants
Source: Cell Mol Life Sci. 2024 May 25;81(1):235. doi: 10.1007/s00018-024-05243-x (PMC11127902; doi:10.1007/s00018-024-05243-x)
Supplement: Supplementary file 1 — Supplementary file1 (DOCX 296 KB) [file 18_2024_5243_MOESM1_ESM.docx]

**Supplementary text**

*GRIN2B*_CRISPR design

>chr6:135740430-135741659 (reverse complement)

CAAGCAGGGAACATAGCTAAAATAACATGTGAATTATCCAATAGCTGGTTTAGGATCAACAGAATTTTG

AGTGCTTTGAAGGTCAAAAGAAAATTTCTTAGCAAAATATATTCAATATTCAAACATTATTTCCATGAC

TGACATGCTGTGTGACCTTAATTGTAGAAGGACAATGTGATTGGGCCTCTGAATTAAGAGGCCTGAGTG

CAGATTTTAGACAATTTTTGGAAATAAAAGTGACTAATGTACCACAAACTATATCATGCCACTTTCAGG

CCCATCATCACTGGTATTGTGTCGGATGCATAGAAGGAACACACAGGGTTACAGATTAAACTCGGGACT

GGGCAAATAGAAATCTCTCAACAATACGTATTAAATTGAGTTGAACTGAAGTCCACTTCCAGAAGGCAA

TTACTGCTCCTGAGTGAGGGCTAATGTGTTTGGTGAATTATAGTCGGTTCCTTTCCATTATAAACTCAC

TAATGCTGACTTAATGTCTCTTCACATTTTTCTAAAGCATTTCTTCTTTAT[TCTGTCTCTGTCTCTCT

CTCTCTGTCTTTCTTTCTCTCTCCAGttccagagacctaatgacttctcaccccctttccgctttggga

ctgtgcccaatggcagcacagagaggaatatccgtaataactatgcagaaatgcatgcctacatgggaa

]agttcaaccaaaggggtgtagatgatgccttgctctccctgaaaacaggGTAAGAACGGCCTCCAAGC

TCAGAGTGATACACCTCTCACATCCACTGGACACAGTTTGAACTTGAACCATACTCATTAAATCAAAAG

ACTGTTTCCAAGTCAAATTTGATGGCATTATTCCAACCCTTGTACAAGAGTTCTTATGATGTTACTTGC

TTCTATATACCATCCTGCAAATTGTTTTCTGCTTGCATGAATGTGGGCATGGAGTGGAAAAGAAACAGA

TCAAAAACTAAAAAGGAAGCGTCAAGGAGAGAGCGCATATCTGTTACACATGAGCTGTAGTCTAAGAGGTGTAATAGACAGTTCCGGTGTCTAGGGAGAAATGAAACAGGAAGTGTGCAAAATTTGTAGACGGCAACAAGTAGAAATGTGTTGATTGATCCCCAATTTCAGCTTTGATTGTCACTCCTGATAGCCATTCTCTGTACC

CAGAGTGTATTTGTTCACAAGCTGTTAATGCAGCGATTTGGGCACACTTGTCCTTTAGT

Phenocopy human mutation G689C G>T

>chr6:135740960-135741093 (reverse complement) TCTCTCTCCAGttccagagacctaatgacttctcaccccctttccgctttgggactgtgcccaatggca gcacagagaggaatatccgtaataactatgcagaaatgcatgcctacatgggaaagttcaaccaa

Guide sequence: CACAGTCCCAAAGCGGAAAG

Repair oligo, Glycine to Cysteine:

KpnI: ggtacc

CTCTGTCTTTCTTTCTCTCTCCAGttccagagacctaatgacttctcaccaccttttcgcttcggtacc

gtgcctaattgcagcacagagaggaatatccgtaataactatgcagaaatgcatgcctac

KPNI-Cleaved product size (wt)- 453 bps, mutant: 285/170 bps

GluN2B G689C animal model sequencing

**Sense AAC TCG GGA CTG GGC AAA TA**

**Antisense CCA GTG GAT GAT GTG AGA GGT G**

NO INDELS!

Second trial

Guide 1: /AltR1/rCrG rCrUrU rUrGrG rGrArC rUrGrU rGrCrC rCrArA rGrUrU rUrUrA rGrArG rCrUrA rUrGrC rU/AltR2/

Guide 2: /AltR1/rGrC rCrCrA rArUrG rGrCrA rGrCrA rCrArG rArGrG rUrUrU rUrArG rArGrC rUrArU rGrCrU /AltR2/

DNA (from nine blastocysts) was extracted, amplified, and sent to sequencing.
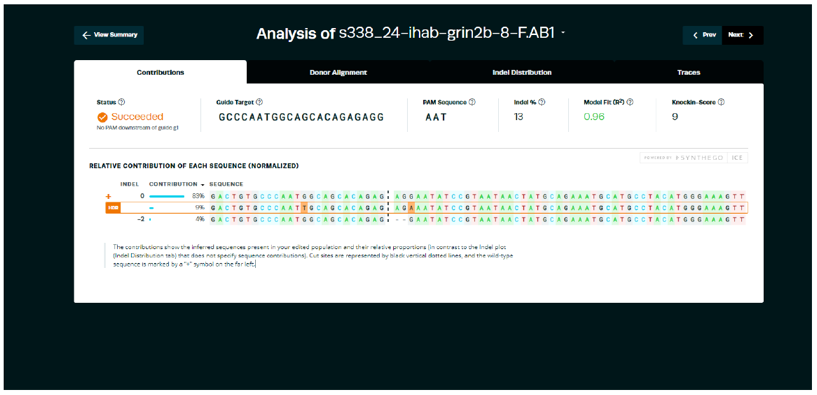

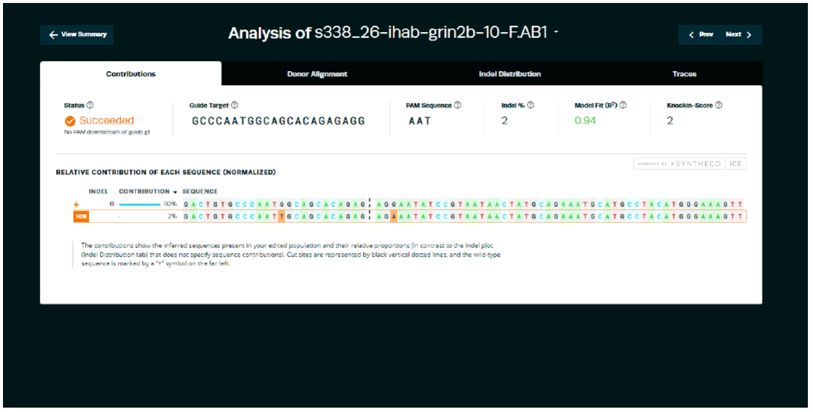

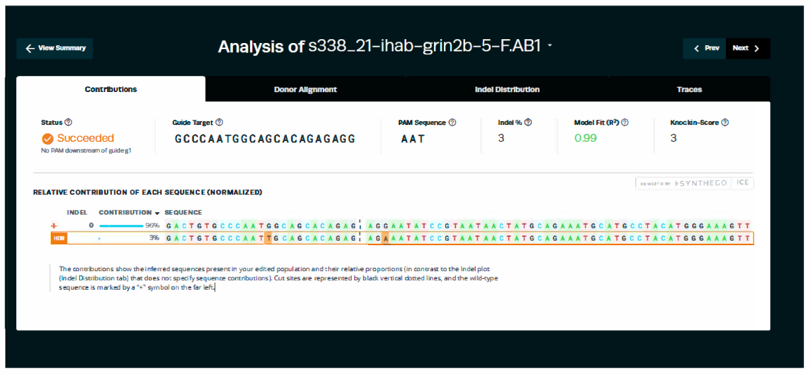


16 pups were obtained, none had insertion/mutation


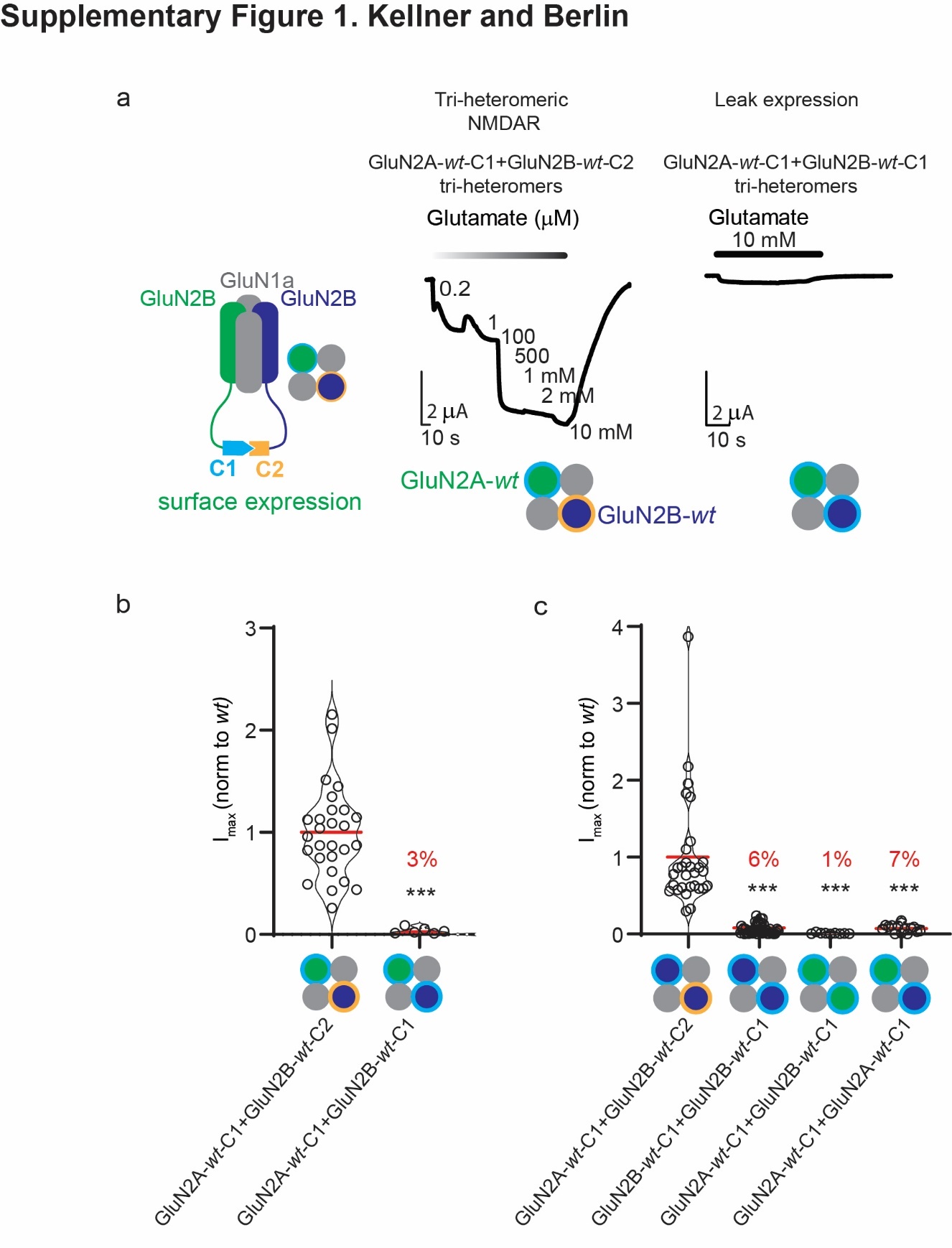


**Supplementary Figure 1. Assessing leak expression for tri-heteromers.**

**a.** Cartoon depiction of a surface expression-enabled tri-heteromeric receptor composed of two GluN1-1a-*wt* subunits (grey) assembled with one GluN2A-*wt*-C1 subunit (green with cyan outline) and one GluN2B-*wt*-C2 subunit (dark blue with orange outline) and representative trace (middle trace). Right panel shows a representative trace of leak tri-heteromeric current recorded from oocytes co-expressing GluN1a-*wt*+GluN2A-*wt*-C1+GluN2B-*wt*-C1. Glutamate (and glycine) application is noted by bars (gradient and black) above traces. **b.** Summary of normalized currents (I_max_) from glutamate dose responses of tri-heteromeric receptors recorded in two independent experiments, 72 hr after mRNA injection; mean is highlighted in red. **c.** Summary of normalized currents (I_max_) from potentiation experiments recorded in two independent experiments, 24 hrs after mRNA injection; mean is highlighted in red. ***, p < 0.001. Significance was assessed by unpaired t-test in **(b)** and Kruskal-Wallis test followed by one-way ANOVA and Dunn’s multiple comparison in **(c).**

**
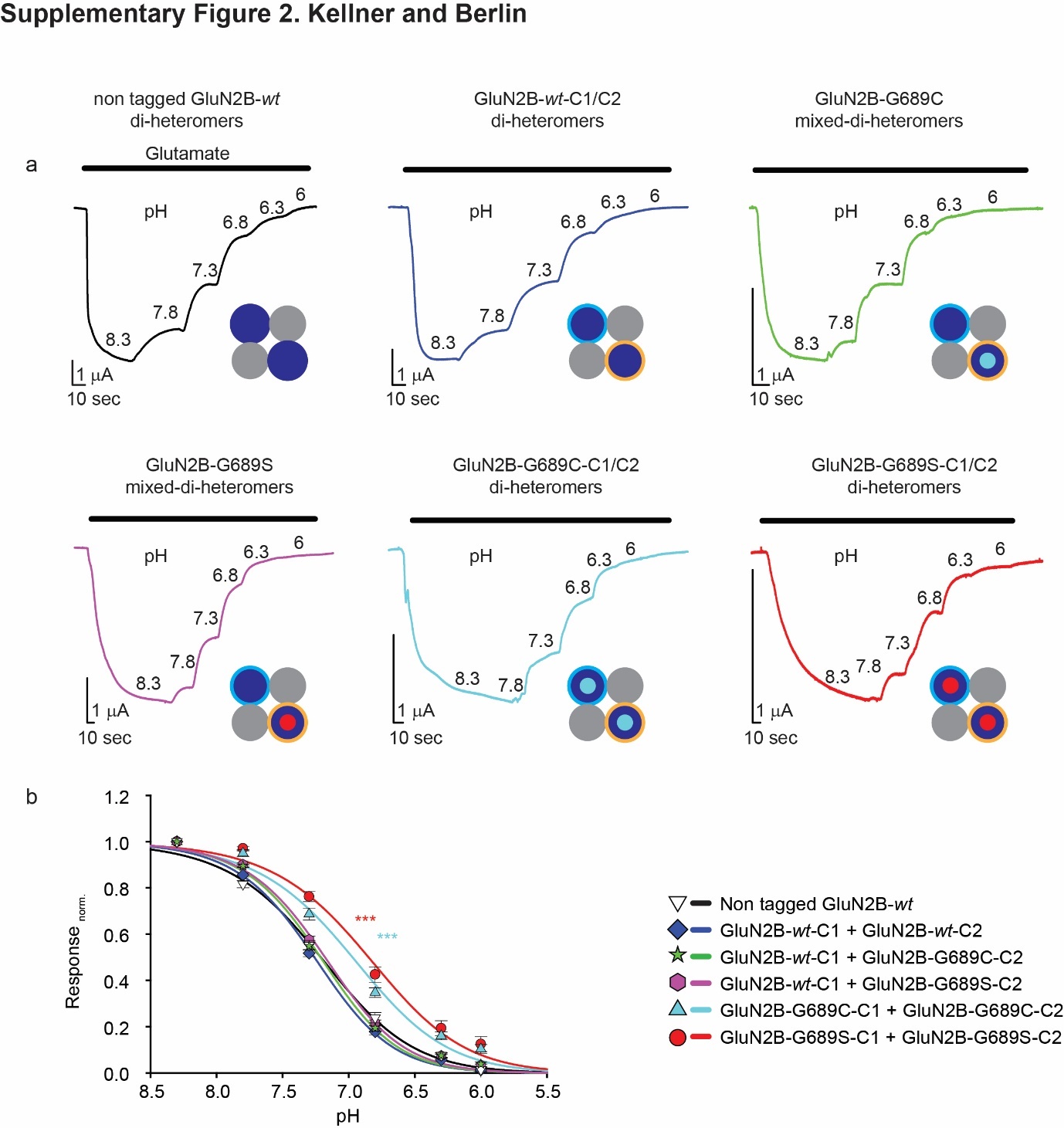
**

**Supplementary Figure 2. GluN2B-variants don’t affect protons sensitivity in *mixed* di-heteromeric receptors.**

**a.** Representative traces from oocytes expressing pure (tagged and non-tagged) or *mixed* GluN2B di-heteromeric NMDARs in response to decrease in pH (indicated next to steps in the trace) in the presence of 5 mM of glutamate and 100 μM glycine (black bars above traces). **b.** Summary of dose-response curves for pure (tagged and non-tagged) and mixed di–heteromeric GluN2B-receptors showing that GluN2B-variants don’t affect protons sensitivity in mixed di-heteromeric receptors; color-coded as in **(a),** Summary of IC_50_ are indicated in Table 1. ***, p < 0.001. Significance was assessed by one-way ANOVA with Tukey post hoc test.

**
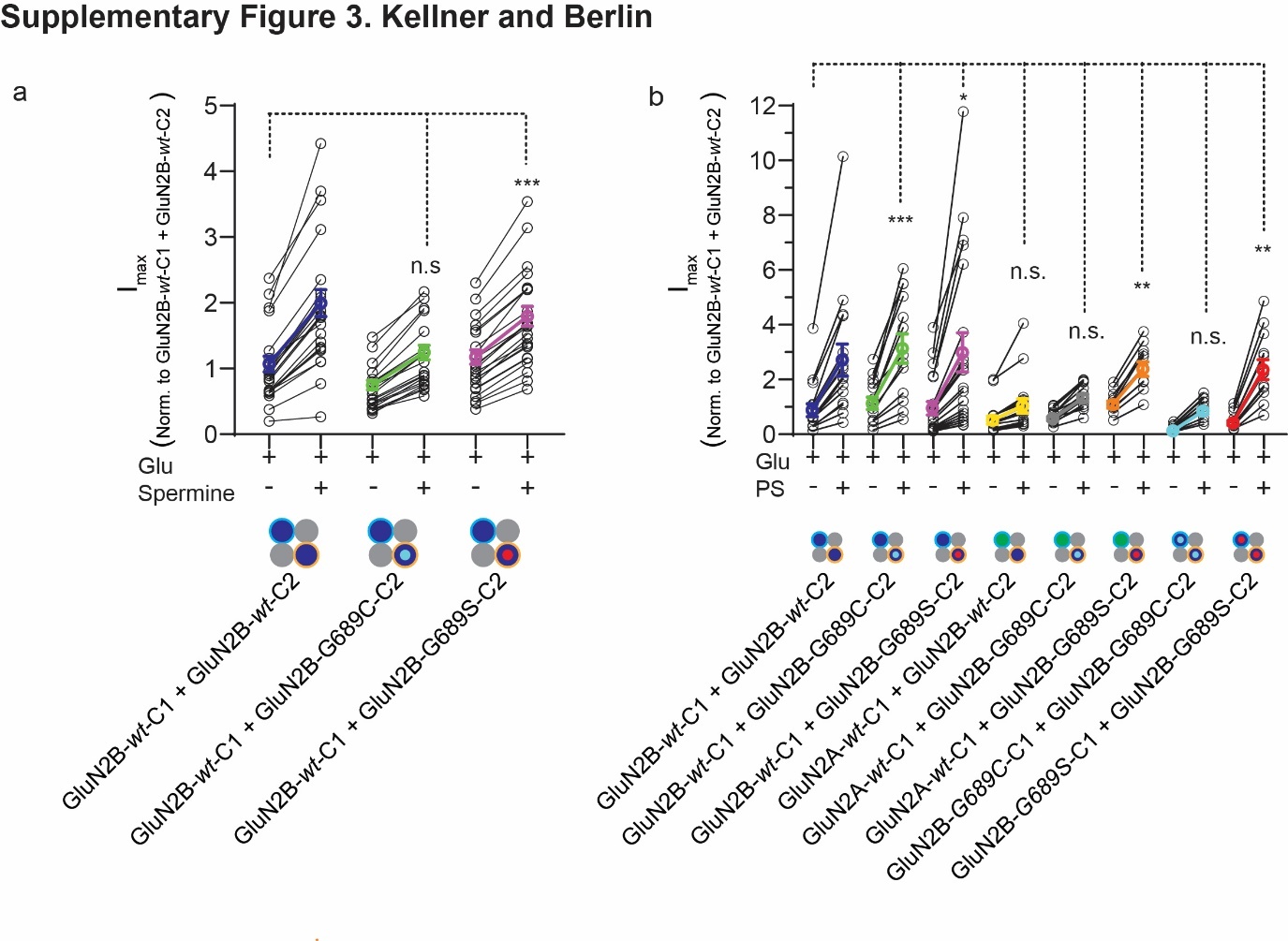
**

**Supplementary Figure 3. Spermine and PS rescue current amplitudes of pure GluN2B-*wt* and mixed GluN2B di-heteromeric receptors expressed in *Xenopus oocytes.***

**a.** Summary of normalized maximal currents (I_max_) of pure GluN2B-*wt* and mixed GluN2B di-heteromeric receptors in individual cells, before and after application of spermine. Spermine enhances the currents of mixed GluN2B-*wt*-C1 + GluN2B-G689S-C2 di-heteromeric receptors currents above the basal current (i.e., I_max_ prior to the application of spermine) of pure GluN2B-*wt* di-heteromers (this group was present in all experiments and served as the reference for all other groups). **b.** Summary of normalized currents of pure and mixed di-heteromeric and tri-heteromeric receptors before and after the application of PS. In both panels, statistical significance was assessed by comparing normalized potentiated current amplitudes of different NMDAR compositions to the I_max_ of purely GluN2B-*wt* receptors prior to the application of the potentiators. n.s., not significant; *, p < 0.05; **, p < 0.01; ***, p < 0.001. Significance was assessed Kruskal-Wallis test followed by Dunn’s multiple comparison post hoc.


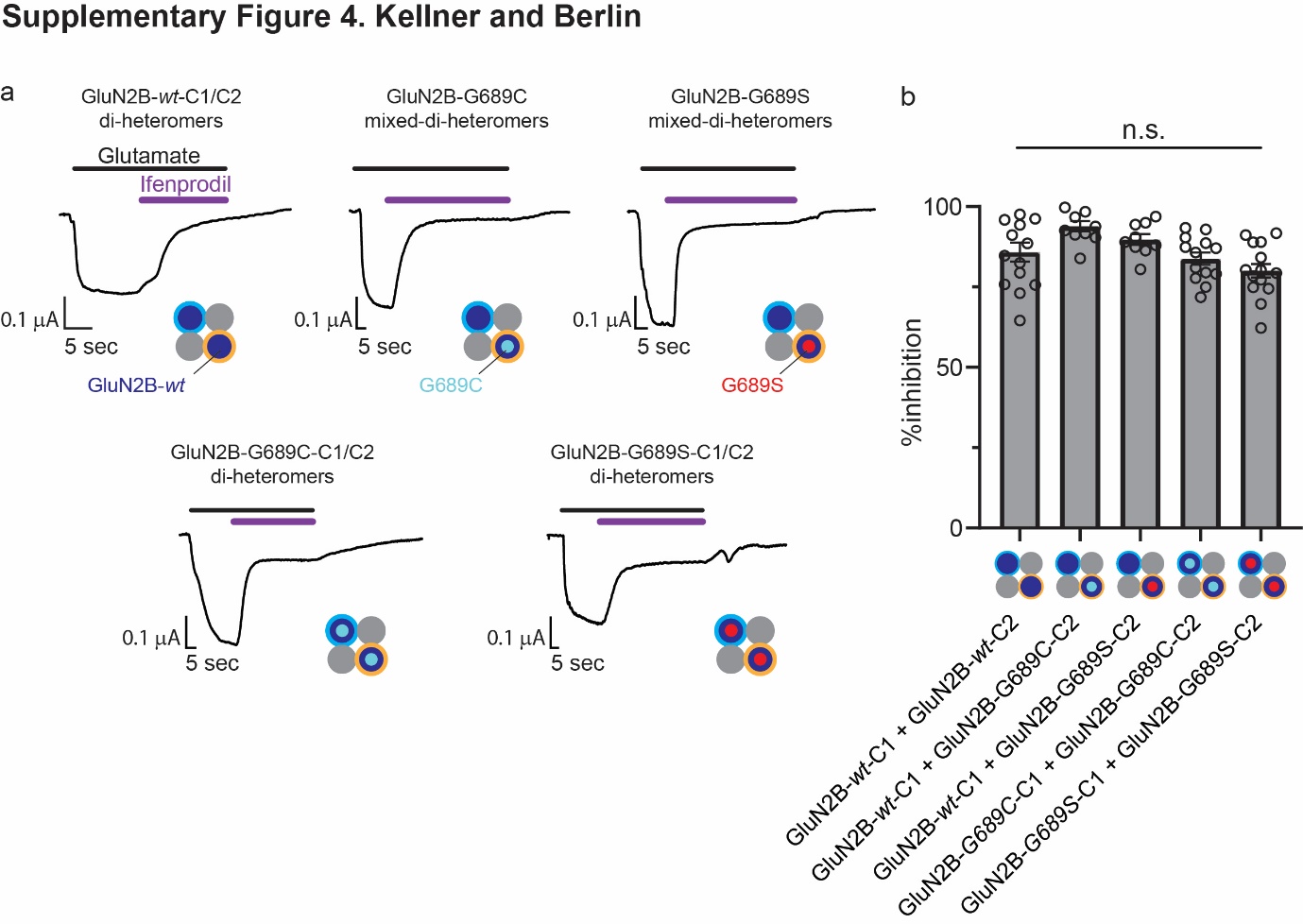


**Supplementary Figure 4. GluN2B variants display similar extent of inhibition by ifenprodil *in xenopus lavies* oocytes**.

**a.** Representative traces from oocytes expressing various receptor subtypes (indicated above traces), activated by 5 mM glutamate and 100 μM glycine (indicated by black bar) and inhibited by 5 μM ifenprodil (indicated by purple bar) in the presence of the agonists. **b.** Summary of the extant of inhibition by ifenprodil over purely variant and mixed di-heteromeric GluN2B channels showing similar extent of inhibition. n.s., not significant; Significance was assessed by one-way ANOVA with Dunnett’s multiple comparison test. For (b) GluN2B-*wt*-C1/C2, N=1, n=14; GluN2B-*wt*-C1-GluN2B-G689C-C2, N=1, n=10; GluN2B-*wt*-C1-GluN2B-G689S-C2, N=1, n=9; GluN2B-G689C-C1/C2, N=1, n=13; GluN2B-G689S-C1/C2, N=1, n=15.

**
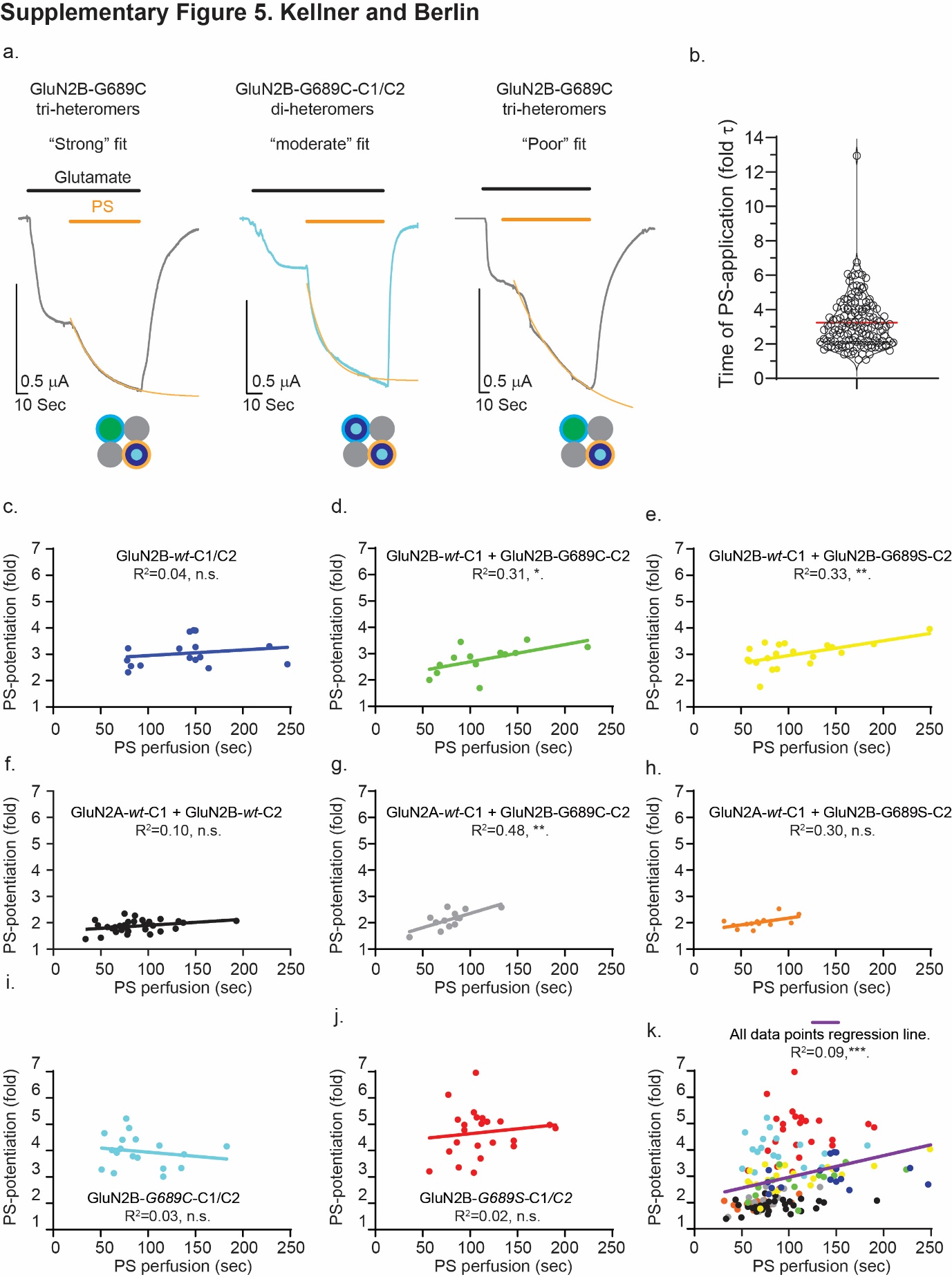
**

**Supplementary Figure 5. Duration of PS application surpasses three time constant (t) of the potentiated current.**

**a.** Representative traces from oocytes expressing various receptor subtypes (indicated below traces), activated by glutamate, and potentiated by PS. The potentiated current was fitted with a standard single-exponential fit (orange fit, see **methods**) from which we extracted the activation time constant (t). Most traces could be fitted faithfully, as shown in left trace (“strong”). In a handful of instances, moderate (middle panel) or poor (right) fits were obtained, suggesting underestimation of τ. **b.** Summary of PS application times with respect to τ (fold). The average application time of PS reflected ~3 times the time constant τ, suggesting that the reaction reached approximately 95% (1 - 1/e^3 ≈ 0.9502) of the steady-state value. Five τ's are needed to reach 99.3% (1 - 1/e^5). **c-k.** Longer perfusion times do not engender larger potentiation (fold). Individual linear regressions of PS-application times vs. PS-potentiation (fold) for each group are shown **(c-j)**, and a combined regression for all groups together **(k)** (bold magenta regression, all data points, color coded as in **c-j**). n.s., not significant; *, p < 0.05; **, p < 0.01; ***, p < 0.001. Significance was assessed by Pearson correlation.

**
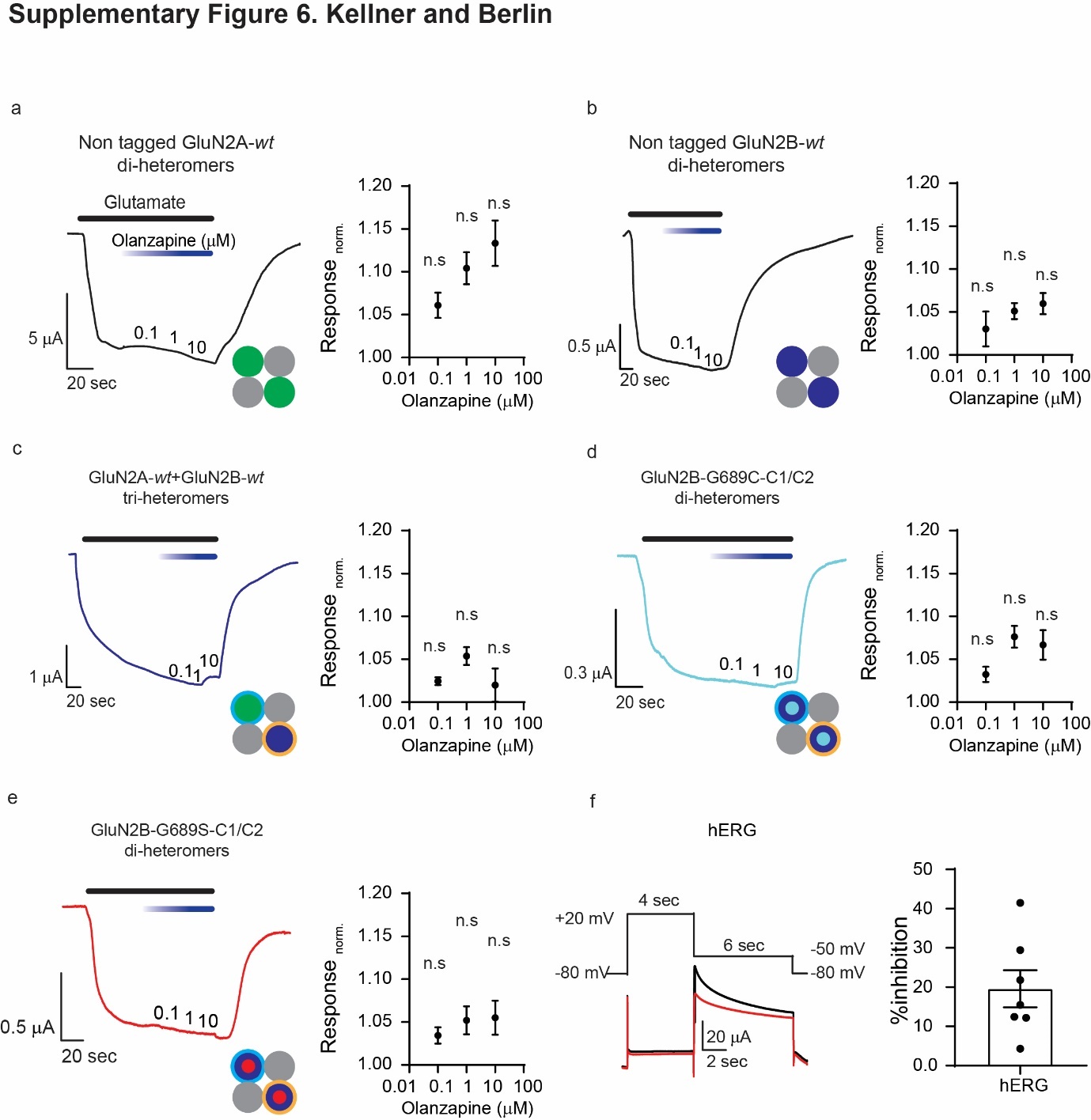
**

**Supplementary Figure 6. Olanzapine does not directly potentiate NMDARs. a-e.** (left) Representative traces from oocytes expressing pure (tagged and non-tagged) di heteromeric or tri-heteromeric NMDARs and their responses to three concentrations of olanzapine (indicated next to current steps on trace in μm) and (right) Summarizes the extent of potentiation of responses normalized to the steady-state current prior to olanzapine application .5mM Glutamate (and 100 μM glycine) application is indicated by black bars, whereas olanzapine application is indicated by gradient dark-blue bars above traces. **f.** Representative trace (protocol noted above trace) from an oocyte expressing the hERG channel, before (black) and after (red) application of 20 μM olanzapine and summary of hERG channels inhibition by olanzapine (right). n.s., not significant. Significance was assessed by one-way ANOVA with Dunnett’s post hoc test. For (a-f) non tagged rat GluN2A-*wt*, N=1, n=11; non tagged human GluN2B-*wt*, N=1, n=11; GluN2A-*wt­-*C1-GluN2B-*wt­-*C2, N=1, n=5; GluN2B-G689C*-*C1/C2, N=1, n=7; GluN2B-G689S*-*C1/C2, N=1, n=9; hERG, N=1, n=7.


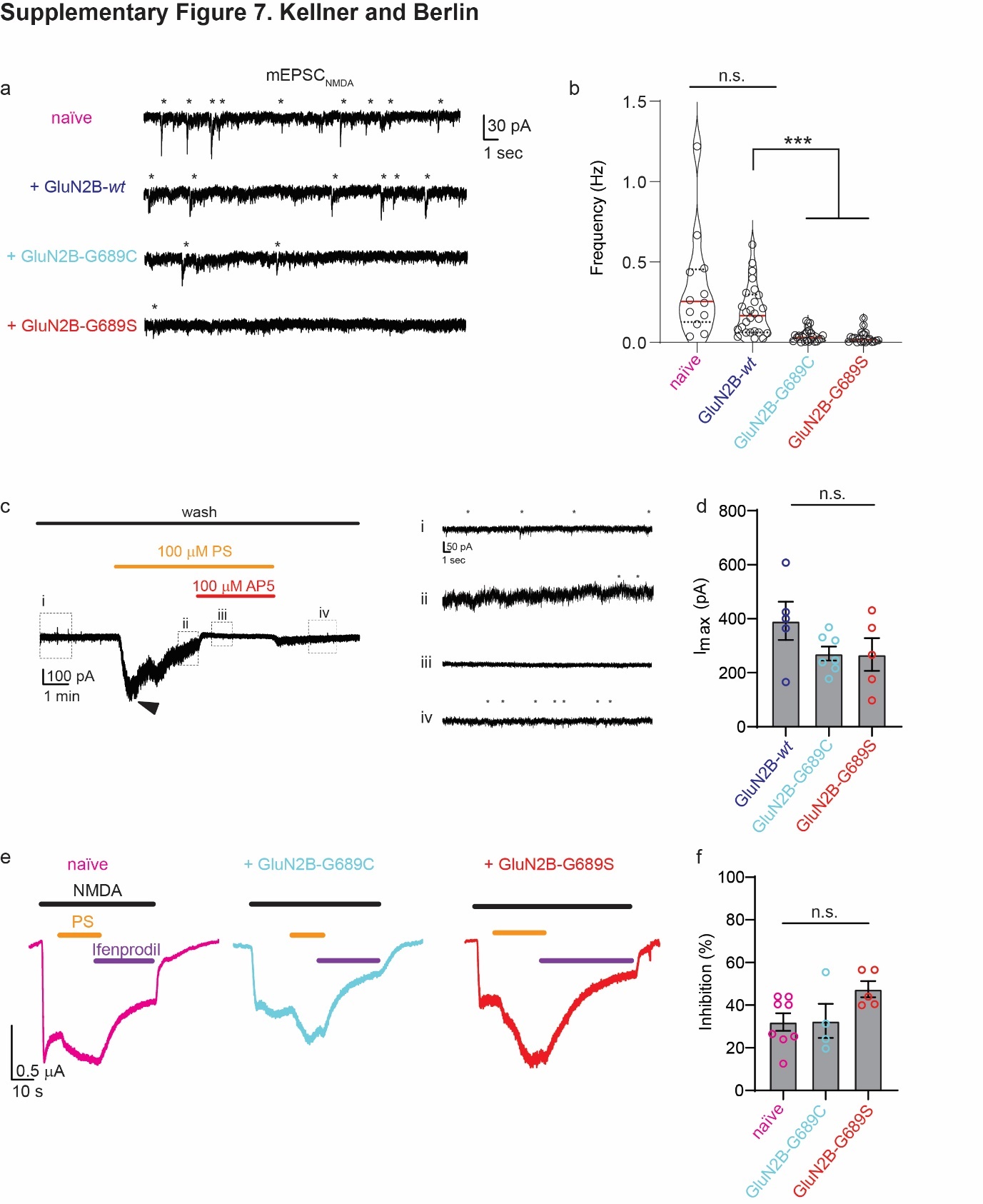


**Supplementary Figure 7. Hippocampal neurons overexpressing GluN2B-variants exhibit diminished synaptic activity.**

**a.** Representative NMDARs-dependent miniature EPSCs (mEPSC_NMDA_) recordings from non-transfected neurons (naïve) vs. neurons transfected with GluN2B-*wt,* GluN2B-G689C or GluN2B-G689S; showing reduced mEPSC_NMDA_ frequency in neurons overexpressing the different variants, summarized in (**b**)**. c.** Effect of PS on mEPSC_NMDA_. Representative recording from a neuron overexpressing GluN2B-*wt*. Following three minutes of recording in wash solution (**Methods**), 100 μM PS was applied (orange bar), giving rise to a large NMDAR-dependent current (maximal response denoted by arrowhead). After three minutes, 100 μM AP5 was co-applied with PS (red bar), then both drugs were washed out (for three minutes). Insets (i-iv) show examples for each state. **d.** Summary of the maximal PS-current for the three groups. **e.** Whole cell recordings of NMDAR-dependent currents from naïve (pink), GluN2B-G689C-transfected (cyan) or GluN2B-G689S-transfected neurons (red) in response to 100 μM NMDA (and 50 μM glycine) and 100 μM PS followed by 2.5 μM ifenprodil (magenta bar). The GluN2B-dependent fraction of the current was determined by application of 2.5 μM ifenprodil in the presence of agonists. **f.** Summary of the degree of inhibition ( in %) induced by application of 2.5 μM ifenprodil at naive and GluN2B-variant over-expressing neurons. n.s., not significant; ***, p < 0.001. Significance was assessed by Kruskal-Wallis test followed by Dunn’s multiple comparison test (**b**) and one-way ANOVA with Dunnett’s multiple comparison test in (**d**) and (**f**). For (b), naïve, N=3, n=12; GluN2B-*wt*, N=3, n= 26; GluN2B-G689C, N=3, n= 23; GluN2B-G689S, N=3, n= 20. For (d) GluN2B-*wt*, N=1, n= 5; GluN2B-G689C, N=1, n= 7; GluN2B-G689S, N=1, n= 5. For (f) GluN2B-*wt*, N=1, n= 7; GluN2B-G689C, N=1, n= 4; GluN2B-G689S, N=1, n= 5.

**
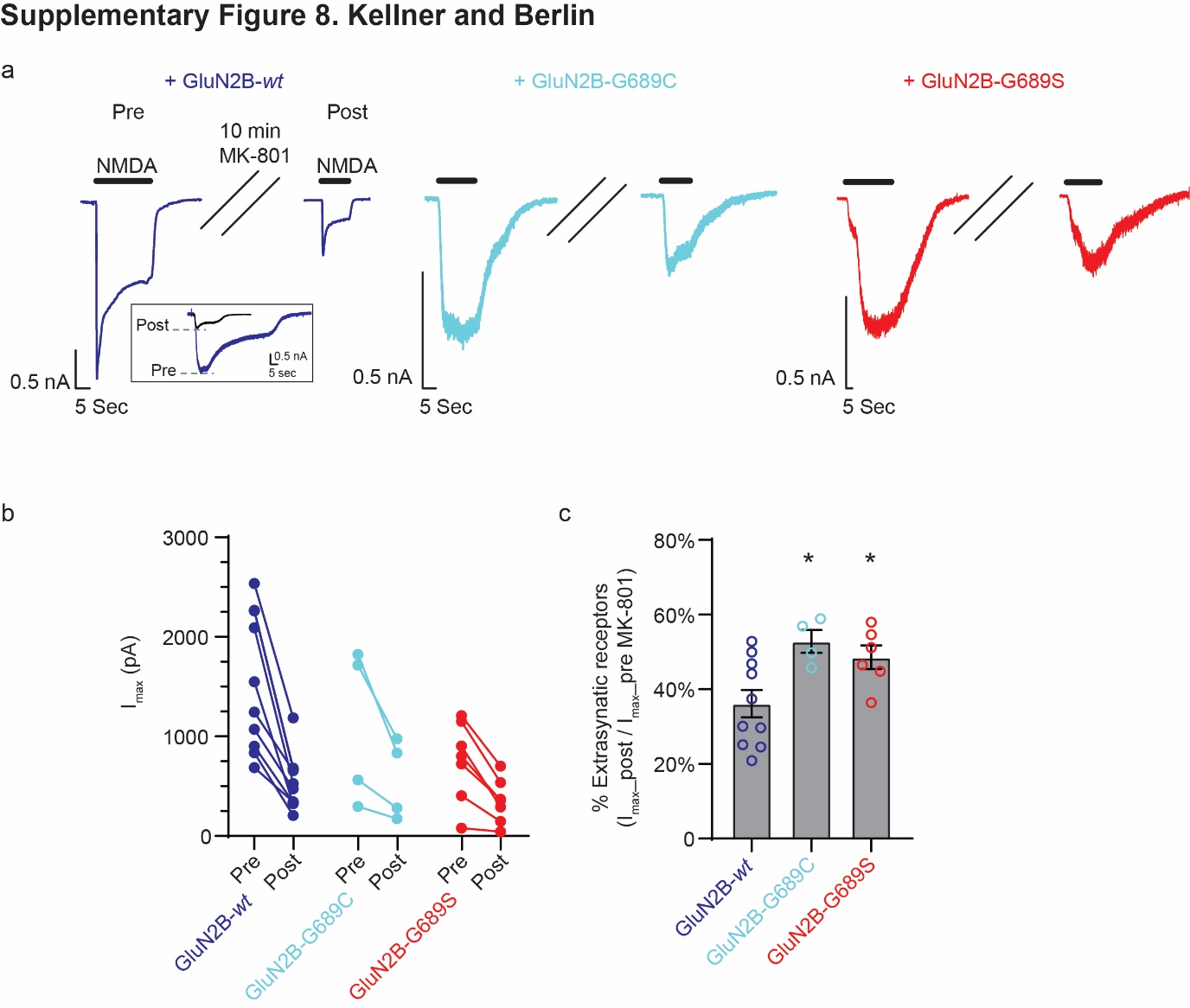
**

**Supplementary Figure 8. GluN2B-variants accumulate in synaptic sites in hippocampal neurons.**

**a.** Representative recordings from hippocampal neurons transfected with GluN2B-*wt* (left panel, dark blue trace), GluN2B-G689C (middle panel, cyan trace) or GluN2B-G689S (right panel, red trace). For assessing extrasynaptic receptors, NMDA was applied before and after 10 minutes application of 1μM MK-801 (indicated above left trace) (see **Methods**). **b.** Summary of the maximal currents (I_max_), before and after application of MK-801. Each bullet represents an individual neuron. **c.** Summary of the fraction of extrasynaptic NMDARs . n.s., not significant; *, p < 0.05. Significance was assessed by one-way ANOVA with Dunnett’s multiple comparison test. For (b) and (c) GluN2B-*wt*, N=1, n= 10; GluN2B-G689C, N=1, n= 4; GluN2B-G689S, N=1, n= 6.

**
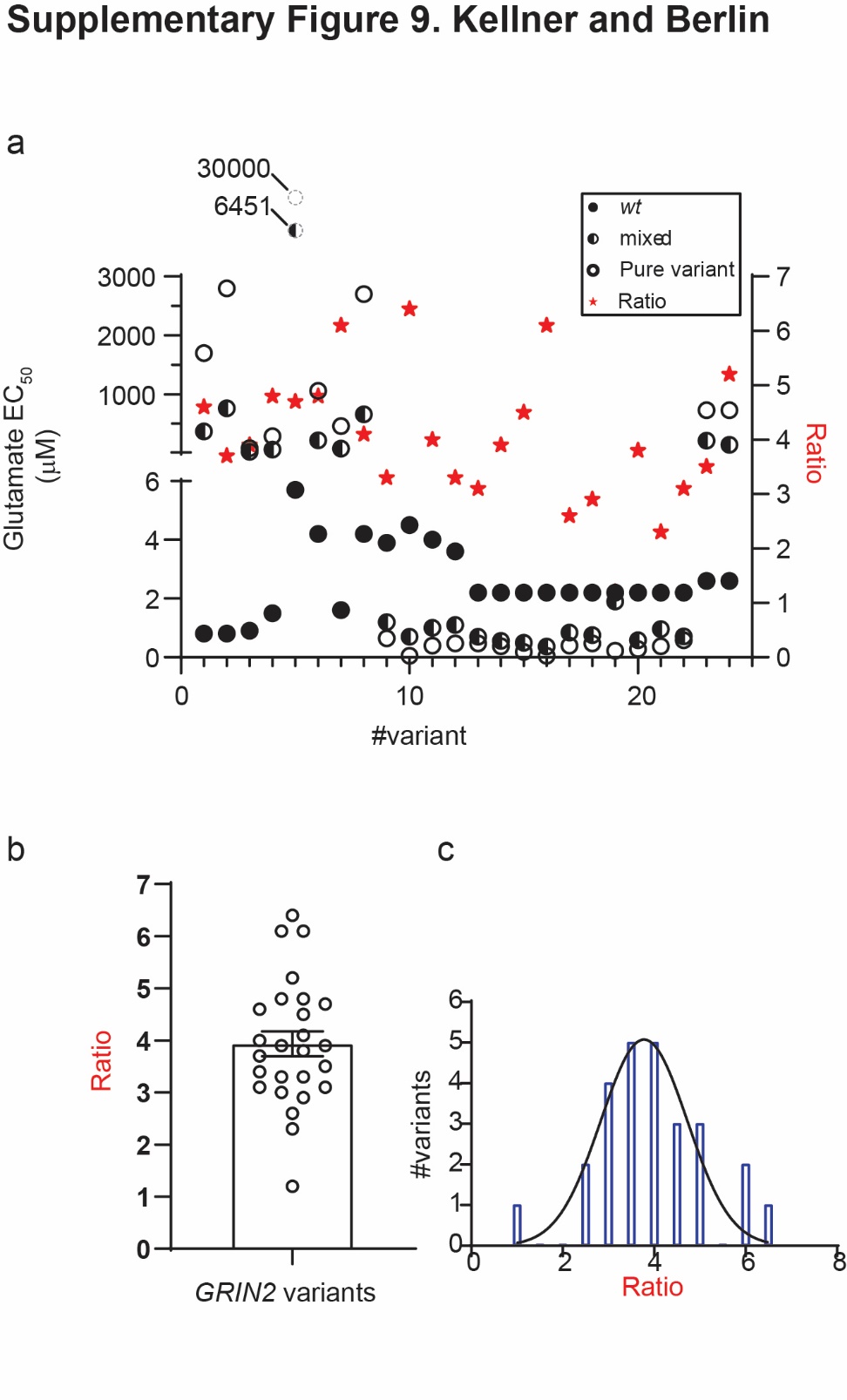
**

**Supplementary Figure 9. Glutamate potency ratio remains constant between lowest affinity composition and mixed di or tri-heteromers.**

**a.** (left Y-axis) Glutamate potencies (EC_50_) for different variant incorporated NMDRs compositions: *wt* di-heteromeric receptors (filled circle), mixed di or tri-heteromeric receptors containing a single GluN2 variant subunit (half-filled circle), and purely variant di or tri-heteromeric receptors (open circle). (right Y-axis) Potency ratio values of each variant (indicated by red stars). Potency Ratio was calculated as: Highest EC_50_/[mixed di or tri-heteromeric EC_50_].(See **Table 2**  and **methods**). X-axis represents each individual *GRIN* variant explored within the three different channel compositions described above (filled, half and empty circles) and the corresponding potency ratio value (red stars) **b.** GRIN2 variants’ potency ratio reveals a robust behavior across 26 variants explored, centering around 4. **c**. Potency ratio is normally distributed.
